# Supplementary material for: Systematic review of metrics used to characterise dietary nutrient supply from household consumption and expenditure surveys
Source: Public Health Nutr. 2022 Jan 13;25(5):1153–65. doi: 10.1017/S1368980022000118 (PMC9991734; doi:10.1017/S1368980022000118)
Supplement: Supplementary file 1 [file S1368980022000118sup.zip › S1368980022000118sup001.docx]

Title: Systematic review of metrics used to characterize dietary nutrient supply from household consumption and expenditure surveys

Shortened Title [38/45 characters]: Review of HCES nutrient supply metrics

Kevin Tang^1 2 *^, Katherine P Adams^3^, Elaine L Ferguson^1^, Monica Woldt^2 4^,

Jennifer Yourkavitch^2 5^, Sarah Pedersen^6^, Martin R Broadley^7^, Omar Dary^8^, E Louise Ander^7 9^, Edward J M Joy^1^

^1^ Department of Population Health, London School of Hygiene & Tropical Medicine, London, WC1E 7HT, UK

^2^ USAID Advancing Nutrition, 4^th^ Floor, 2733 Crystal Drive, Arlington, VA, 22202, USA

^3^ Institute for Global Nutrition, University of California, Davis, One Shield Avenue, Davis, CA, 95616, USA

^4^ Helen Keller International, 4^th^ Floor, OAS Building, 1889 F Street, NW, Washington, DC, 20006, USA

^5^ Results for Development, 1111 19^th^ Street, NW, Suite 700, Washington, DC, 20036, USA

^6^ USAID, Bureau for Resilience and Food Security, 1300 Pennsylvania Avenue, NW, Washington, DC, 20523, USA

^7^ School of Biosciences, University of Nottingham, Sutton Bonington, Loughborough, LE12 5RD, UK

^8^ USAID, Bureau for Global Health, 1300 Pennsylvania Avenue, NW, Washington DC, 20523, USA

^9^ Centre for Environmental Geochemistry, British Geological Survey, Keyworth, Nottingham, NG12 5GC, UK

*Corresponding Author

Kevin Tang

Department of Population Health

London School of Hygiene & Tropical Medicine

Keppel Street

London, WC1E 7HT, United Kingdom

P: (+44) 20 7636 8636

F : (+44) 20 7436 5389

Email: [kevin.tang1@lshtm.ac.uk](mailto:kevin.tang1@lshtm.ac.uk)

**FINANCIAL SUPPORT**

This work was supported by the United States Agency for International Development (USAID) through its flagship multi-sectoral nutrition project, USAID Advancing Nutrition under the terms of contract 7200AA18C00070 awarded to JSI Research & Training Institute, Inc. This work was also supported, in part, by the Bill & Melinda Gates Foundation [INV-002855]. Under the grant conditions of the Foundation, a Creative Commons Attribution 4.0 Generic License has already been assigned to the Author Accepted Manuscript version that might arise from this submission. The contents of this article do not necessarily reflect the views of USAID, the U.S. Government, or the Bill & Melinda Gates Foundation.

**ACKNOWLEDGEMENTS**

We are deeply grateful for the contributions from Frances Knight (London School of Hygiene & Tropical Medicine/World Food Programme), Alberto Zezza and Sydney Gourlay (both from the World Bank Living Standard Measurement Study) in the development of this analysis. We thank Fanny Sandalinas (London School of Hygiene & Tropical Medicine) and Lucia Segovia De La Revilla (London School of Hygiene & Tropical Medicine) for their review and technical assistance. For additional support, we thank Elaine Borghi (World Health Organization), Saskia De Pee (World Food Programme), Keith Lividini (International Food Policy Research Institute/Harvest Plus), Kristen MacNaughten (Bill & Melinda Gates Foundation), Ana Moltedo (Food & Agriculture Organization), Lisa Rogers (World Health Organization), Juan Pablo Peña-Rosas (World Health Organization), and Becky Tseng (Food Fortification Initiative).

**CONFLICT OF INTEREST**

There are no potential conflicts of interest relevant to this article.

**AUTHORS CONTRIBUTIONS**

K.T., M.R.B., E.L.A., and E.J.M.J. contributed to the conception and design of the study. K.T., K.P.A., E.L.F., M.R.B., and E.J.M.J. contributed to the development of the original study protocol. All authors participated in the analysis and deductions of paper. K.T. identified existing literature via the online database search and snowballing. K.T. and E.J.M.J. reviewed literature to evaluate for inclusion. K.T. extracted the data from the original manuscripts and prepared the tables and figures. All authors contributed to the interpretation of the data, critically reviewed the manuscript for important intellectual content and approved the final manuscript.

**ETHICAL STANDARDS DISCLOSURE**

Ethical approval for this study was provided by the London School of Hygiene & Tropical Medicine Observational Research Ethics Committee (Reference 21903).
