# Supplementary material for: Systematic review of metrics used to characterise dietary nutrient supply from household consumption and expenditure surveys
Source: Public Health Nutr. 2022 Jan 13;25(5):1153–65. doi: 10.1017/S1368980022000118 (PMC9991734; doi:10.1017/S1368980022000118)
Supplement: Supplementary file 1 [file S1368980022000118sup.zip › S1368980022000118sup002.docx]

**ABSTRACT** [Word count: 239/250 max]

**Objective:** To review existing publications using Household Consumption & Expenditure Survey (HCES) data to estimate household dietary nutrient supply to (1) describe scope of available literature, (2) identify the metrics reported and parameters used to construct these metrics, (3) summarize comparisons between estimates derived from HCES and individual dietary assessment data, and (4) explore the demographic and socioeconomic sub-groups used to characterize risks of nutrient inadequacy.

**Design:** This study is a systematic review of publications identified from online databases published between 2000 to 2019 that used HCES food consumption data to estimate household dietary nutrient supply. Further publications were identified by “snowballing” the references of included database-identified publications.

**Setting:** Publications using data from low- and lower middle income countries

**Results:** In total, 58 publications were included. Three metrics were reported that characterized household dietary nutrient supply: apparent nutrient intake per adult-male equivalent per day (n=35), apparent nutrient intake per capita per day (n=24), and nutrient density (n=5). Nutrient intakes were generally overestimated using HCES food consumption data, with several studies finding sizeable discrepancies compared to intake estimates based on individual dietary assessment methods. Sub-group analyses predominantly focused on measuring variation in household dietary nutrient supply according to socioeconomic position and geography.

**Conclusion:** HCES data are increasingly being used to assess diets across populations. More research is needed to inform the development of a framework to guide the use of and qualified interpretation of dietary assessments based on these data.

# INTRODUCTION

Vitamins and minerals, also known as micronutrients, are required in small quantities in the diet, and are essential for human health ^(1)^. Micronutrient deficiencies continue to burden billions of people worldwide, disproportionately affecting the world’s poorest populations ^(2)^. Poor quality diets, among other interconnected risk factors, are a main cause of micronutrient deficiencies because individuals do not consume adequate quantities of bioavailable nutrients to meet their physiological requirements ^(3)^. Diet-related risk factors are compounded by other systemic social issues common in poor populations, such as inadequate healthcare, high rates of infection and poor sanitation ^(4,5)^, and a lack of education ^(6–8)^. As highlighted in the 2020 Global Nutrition Report, reducing micronutrient deficiencies will require a coordinated effort between governments, businesses and civil society to address deep inequities that arise from unjust systems and processes, particularly for the world’s most nutritionally vulnerable groups ^(9)^.

Quantifying and characterizing national burdens of micronutrient deficiencies requires a combination of data types. These include biomarker assessment in micronutrient surveys ^(6,10)^, estimates of national micronutrient supply in food systems ^(11)^, and individual micronutrient intake estimates from food consumption surveys ^(12)^. However, in many contexts, these data require significant time, capacity, expertise and specialist equipment to collect and analyze, meaning few countries routinely collect this data at scale. Household Consumption and Expenditure Surveys (HCESs) can contribute a unique source of nationally representative food acquisition data that are regularly collected in low- and lower-middle income countries ^(13)^. Questions about households’ food consumption and expenditures using a country-specific food item list are commonly integrated into HCESs and this data can be used to estimate the acquisition and apparent consumption of foods by members of the household ^(14)^.

HCES is a term used to refer to a family of nationally and sub-nationally representative, multicomponent surveys (e.g. Household Income and Expenditure Surveys, Socioeconomic Surveys, Living Standards Measurement Surveys, etc.), which are primarily designed to provide data to characterize an array of socioeconomic conditions. National HCESs are implemented by national statistical agencies, often with technical assistance from the World Bank’s Living Standards Measurement Study group. In most countries, bar a few exceptions, this data is publicly accessible, and can be used for a variety of rapid analyses, for instance, to monitor poverty, measure economic inequality, characterize vulnerability to economic shock, estimate agricultural production, calculate macroeconomic aggregate indicators (e.g. gross domestic product), and construct consumer price indices ^(15–17)^. While HCES data in some countries have been publicly available since the 1980s, only more recently have data from the household food supply modules of these surveys been used to estimate individual-level micronutrient intake and to inform public health research and decision-making ^(18)^. Alongside increased use of HCES food acquisition data has come an interest in assessing the how well these data can proxy for individual dietary assessment data^(19)^.

HCES data are subject to error when used to estimate individual-level food consumption, which raises challenges for their use in estimating nutrient intakes^(19,20)^. Due to this, the use of the terms “nutrient supply” at the household level and “apparent nutrient intake” at the individual level are used to reflect the sources of error and imprecision when estimating nutrient intake from HCES compared to other, more direct nutritional assessment methods (Table 1). Sources of potential error include assumptions about the intrahousehold food allocation, shortcomings in HCES questionnaire design related to food consumption (e.g. missed foods in pre-defined list of food items/groups, capturing composite foods using recipes, food waste, foods consumed away from home, collecting data only on food acquisitions rather than acqusitions and consumption), recall bias due to longer recall periods, and estimating quantities consumed^(21)^. While most HCESs collect both food consumption (total quantity of foods consumed) and food expenditures (total monetary amount spent on consumed foods), some HCESs only collect food expenditure data, which requires conversion of monetary units into units of food mass (e.g. grams) before calculating household nutrient supply ^(13)^. In addition, errors may be context specific, as HCES methods vary between countries, introducing a differential bias when making comparisons across populations. Recent reviews ^(13)^ and guidelines ^(22)^ have identified differences between HCES questionnaire designs from different countries, with the authors calling for analyses to evaluate the effect of these differences on dietary nutrient supply estimates. Subsequent analyses have reported potential effects due to the following: recall period length (7 to 30 days) ^(23)^, measuring foods consumed away from home ^(24)^, food acquisition compared to consumption ^(25)^, and length and composition of food item lists (50 to 300 items) ^(26)^.

Despite these potential limitations, HCES remain a source of information on what people eat and there is an expanding literature of studies that use HCES to estimate dietary micronutrient supplies and risks of deficiency. Studies have employed a variety of different metrics to report estimates of dietary nutrient supply/apparent nutrient intake and the prevalence of inadequacy, each requiring specific interpretation. This systematic review aims to identify and describe existing publications using HCES data to estimate nutrient supply/apparent nutrient intake in order to document current practices as they relate to the analysis of HCES food consumption data, summarize results of publications that aimed to validate the use of HCES consumption data to estimate nutrient supply/apparent nutrient intake, and provide insights into how these estimates can identify sub-groups at heightened risk for micronutrient deficiencies from dietary inadequacy. This analysis addresses the following questions:

1. What is the scope of the existing literature?
2. What metrics have been used to estimate nutrient and energy supply/apparent intake and nutrient adequacy from HCES data, and how do those estimates compare across metrics?
3. How do HCES-derived estimates of apparent nutrient and energy intakes compare to those estimated using individual-level dietary assessment methods? Do results differ by age group (i.e. adults, adolescents, children, infants)?
4. What types of sub-group comparative analyses have been conducted using nutrient and energy supply/apparent intake estimates from HCES data?

# METHODS

We conducted a systematic review following the Preferred Reporting Items for Systematic Reviews and Meta-Analyses (PRISMA) guidelines ^(27)^. The study protocol is provided in Appendix 1 and was registered with the PROSPERO international database for systematic reviews (ref. 223928).

## Identification and screening of literature

A two-step procedure was used to identify and screen the available literature. First, 14 public databases were systematically searched by one author in the domains of public health (e.g. Global Health Database, PubMed), agriculture and food systems (e.g. AgEcon, AGRIS, GARDIAN CGIAR, IFPRI Publication Database), economics (e.g. EconLit) and general academia (e.g. Google Scholar, JSTOR, SCOPUS, Web of Science). The primary search terms were the different HCES name variations in both English and French as listed by Fiedler *et al.* ^(21)^. Where HCES search terms returned a large number of results (>1000), secondary search terms were used to describe the food consumption module of the HCES questionnaire (e.g. “nutrition”, “consumption”, “food”, “micronutrient"). The search terms for all databases used in this review are included in Appendix 2. Second, the reference list of all included publications identified through the database search were systematically searched to identify additional papers not identified by the database search ^(28)^. Titles and abstracts of all returned literature were screened to identify analyses using the food consumption module of the HCES, where positive screens were included for full-text review.

## Eligibility, inclusion and exclusion criteria

Literature selected by the identification and screening procedure underwent a full-text review for inclusion into this study independently by two authors. Literature was included if it: (1) used HCES data (or other household food consumption data using an HCES questionnaire format) from a low- or lower-middle-income country ^(29)^, and (2) estimated household nutrient supply or apparent intake of nutrients or energy using data from the HCES food consumption and/or expenditure module. Literature was excluded if it: (1) was written in a language other than English or French, or (2) was published before the year 2000. Any disagreements between the two co-authors about whether to include a publication were resolved through discussion. Data were managed using Mendeley Desktop (version 1.19.4; Elsevier, London, United Kingdom).

## Data extraction

Extraction of information from each publication was archived for comparison in Microsoft Excel (version 16.36; Microsoft Corp, Redmond, USA) by one author. A complete list of all categories and extracted information is provided in Appendix 3. In brief, for Question 1, extracted data included the country/year the HCES was implemented, the nutrients analyzed, recall period, methods of processing the HCES data, and food composition data used. For Question 2, key information included the reported nutrient metric, parameters used for estimating energy requirements, and reference values used to define inadequacy. For Question 3, key information included the individual dietary assessment method used if HCES-derived apparent intake estimates were compared with individual-level nutrient intake estimates, differences in nutrient intake and apparent nutrient intake estimates, and disaggregation by age group if reported. For Question 4, we used the PROGRESS+ framework to guide extraction of information describing stratification by social determinants of diets ^(30)^. This framework, which captures place of residence, race/ethnicity/culture/language, occupation, gender/sex, religion, education, socioeconomic status, and social capital, can be used to apply an equity lens when conducting public health research.

# RESULTS

## Scope of existing literature

In total, 58 publications were identified either by the database search (n=48) or by snowballing (n=10) (Figure 1). A list of these publications, their data sources, and reported metrics is provided in Supplementary Table 1. These publications included data collected from 24 different countries (Figure 2A) where 4 publications focused on more than one country. The highest frequency of analyses was observed for data collected in Bangladesh (n=14 publications), Malawi (n=9 publications), India (n=6 publications) and Uganda (n=6 publications). Household dietary energy supply was reported by 83% (n=48) of publications, where 33% (n=19) reported only dietary energy supply without reporting nutrients. Of the publications that did report nutrient supply/apparent intake, estimates covered 20 different nutrients (Figure 2B). The most common nutrients reported were iron (n=32 publications), vitamin A (n=32 publications), and zinc (n=27 publications). The number of publications per year increased substantially after 2012 (Figure 3).

Characteristics of food consumption data used in the included publications are provided in Supplementary Table 2. The term household food “consumption” was reported in 60% (n=35) of publications, whereas 41% (n=24) used a number of other verbs to report the quantity of food in the survey questionnaire (e.g. “acquired”, “purchased”, “received”). More than half of the publications (n=36) used nationally specific food composition tables to estimate nutrient or energy content of foods. In countries where nationally specific food composition tables either did not exist or had missing values, data were drawn from food composition tables of neighboring countries or for the broader region (26%, n=15) or from food composition values of US food items compiled by the US Department of Agriculture or other international food composition databases (38%, n=22). Steps taken to clean and process HCES data varied by country due to differences in survey design, but conversion of food item quantities from local units (e.g. plate, bucket, heap) into standard units (e.g. kilograms), adjustments for edible portions of foods, adjustment for cooking yields, and adjustment of outliers were most frequently reported. However, many studies did not provide sufficiently detailed descriptions of methods to make the study independently repeatable.

## Summary of HCES nutrient supply and adequacy metrics

Three nutrient supply metrics were identified in the included publications (Figure 4). First, the adult male equivalent (AME) approach to estimate apparent nutrient intake was used in 57% (33/58) of the publications. The AME approach assumes that nutrient supplies are distributed among household members in proportion to the energy requirements of each household member, which are standardized in relation to the energy requirement of an adult male ^(31)^. All studies using the AME approach assumed all household members had moderate or moderate-high physical activity levels when estimating individual energy requirements. Second, daily apparent nutrient intake *per capita* was used in 41% (24/58) of publications. *Per capita* adjustment assumes that nutrient supplies are equally distributed among all members within the household (e.g. an adult male and his young daughter are assumed to consume equal food portions). Third, the nutrient density of household diets, or nutrient supply *per* energy supply, was used in 9% (5/58) of publications.

When evaluating inadequacy, *per capita* and AME metrics were used to estimate the population’s adequacy of dietary nutrient or energy supply by comparing apparent intake to a reference nutrient intake (39/58; 67%). Of these publications, 27 focused on micronutrients, where apparent intake estimates were compared to the Estimated Average Requirement (23/27, 85%), the Recommended Nutrient Intake (7/27; 26%), and/or the Recommended Daily Allowance (1/27; 4%) for each micronutrient of interest. Among the 23 publications that focused on dietary energy, 57% (13/23) compared household energy supply to the household’s total energy requirements calculated as the sum of the individual requirements of all household members, and 43% (10/23) compared individual-level apparent energy intake to a pre-defined energy requirement threshold for an adult (e.g. 2100 kcal *per* day). No publications compared nutrient density estimates to nutrient density reference values, such as the critical nutrient density as defined by Vossenaar *et al* ^(32)^. Of all the publications comparing nutrient or energy supply to a dietary requirement to estimate dietary inadequacy, 13% (5/39) included additional nutrient or energy requirements necessary for a pregnancy in the household and 23% (9/39) included additional requirements necessary for women during lactation.

*Comparison of HCES nutrient supply to individual-level dietary intake data*

For publications reporting apparent nutrient intake using the AME approach, six compared their results to individual-level estimates based on a variety of different types of individual-level dietary intake data. This included individual 24-hour dietary recalls of specific demographics within the household (e.g., children under 5 years, women of reproductive age) ^(33–35)^, 24-hour dietary recall of all household members estimated by the meal preparer ^(19,36)^ or observed-weighed food records of specific demographics within the household ^(37)^. Among these publications, four compared nutrient intake/apparent intake estimates from the same households or individuals within these households ^(19,33,36)^ and two compared nutrient intake/apparent intake estimates using different survey populations from the same country ^(34,35)^. Two publications used the same data from the Bangladesh Integrated Household Survey^(19,36)^.

For AME apparent intake estimates, four publications estimated apparent intake per AME using household-level food consumption data recalled over time horizons of 7 to 30 days and compared those to estimates based on individual-level dietary intake data recalled ^(19,33,36)^ or collected prospectively ^(37)^ over 24 hours (Table 2). These studies allow for a true inter-comparison of how well HCES-style nutrient assessments perform compared to 24-hour recalls, which are likely to have lower measurement error. In these studies, AME-based apparent intakes consistently overestimated individual nutrient and energy intakes, and the percentage point difference between the two methods varied according to nutrient or energy and across studies (Table 2: percentage point difference range = 12% to 72%).

Two publications reported sub-group analyses for children under five years old (Table 2) ^(19,37)^. The two publications that used the same household 24-hour dietary recall data to assess intrahousehold food distribution found the largest differences for children under 2 years of age, suggesting greater uncertainty in apparent intake assumptions for infants when compared to other age groups. However, none of the studies incorporated the contributions of energy or nutrient intakes from breastmilk, potentially affecting apparent intake estimates for infant demographics.

One publication compared differences in the nutrient density metric when applied to HCES data versus corresponding 24-hour dietary recall data in Uganda and found no statistically significant difference in median nutrient density for the 14 nutrients assessed with the exception of folate (8% to 26% difference, depending on region), vitamin B_12_ (10% to 93% difference), and vitamin C (19% to 30% difference) ^(38)^. In this analysis, nutrient densities estimated from HCES data were consistently lower than nutrient densities estimated from corresponding 24-hour dietary recall data for almost all nutrients.

## Sub-group nutrient supply and inadequacy comparative analyses

Most publications included in this review stratified nutrient supply estimates by one or more sub-groups of interest. The stratification of nutrient supply by sub-group characteristics was more frequent for indicators of socioeconomic position and geography than for socio-cultural demographic or intra-household characteristics (Figure 5). Indicators of socio-economic position, as defined by Howe *et al.* ^(39)^, included total household income, household expenditure, durable asset ownership, level of educational attainment, and occupation. Geographic characteristics generally reflected HCES sampling methodology, where HCES are designed to be representative of geopolitical administrative regions (n=30) and urban versus rural settings (n=29). A number of publications stratified nutrient supply estimates by individual-level characteristics of members within the household including sex (n=21) and age (n=21), however the definition of age and sex of the household varied. For instance, analyses using only household-level data either used individual characteristics of the “head of household” as a proxy for the entire household (n_sex_=10; n_age_=6) or divided household nutrients according to energy requirements of individuals as a proxy for apparent individual intake of household members (n_sex_=9; n_age_=6). Some HCES data included individual-level food consumption data of household members which enabled the calculation of nutrient intake specific to the sex (n=2) and age group (n=1).

# DISCUSSION

This systematic review found that the use of HCES data to quantify, characterize and evaluate the adequacy of household nutrient supply across populations is being increasingly represented in published literature. Three primary metrics have been used to report dietary nutrient supply. These are (1) apparent nutrient intake using the AME approach to allocate household nutrient supply among constituent members, (2) apparent nutrient intake using the *per capita* approach where household nutrient supply is divided evenly among constituent members, and (3) nutrient density of household diets. Each of these metrics have strengths and limitations when estimating the adequacy of nutrient supply and identifying vulnerable populations at risk of deficiency. While other reviews have analyzed applications of HCES data for broader food security indicators ^(40)^, this is the first review to summarize the use of HCES data to estimate nutrient supply and evaluate adequacy, an increasingly common practice accepted by the nutrition community.

Households differ in size and composition across and within countries and regions, so the majority of publications attempted to convert nutrient supply estimated at the household level to estimates of individual-level apparent intake ^(41)^ using one of two approaches: *per capita* or AME. While the use of the *per capita* approach is simpler, it does not account for any difference in nutrient intakes between household members, which will skew estimates between households of different composition, especially those with large families. Following the publication of a paper series in 2012 ^(3,31)^ that described the methods to distribute nutrients according to AME energy requirement and its application to household micronutrient supply, an increasing number of publications have estimated dietary supply using the AME approach. The major assumption underlying this approach is that foods consumed by the household are distributed among all household members in proportion to each member’s energy requirements, but the method requires data describing individual household members’ age, sex, weight, physical activity level, pregnancy status and lactation status ^(31)^. HCESs often collect data on individual household members’ age and sex, but data on other parameters affecting individual energy requirements are often not available and require simplifying assumptions to calculate the AME factors (e.g. assume a moderate physical activity level for all members of all households). With 57% of publications identified in this review estimating apparent nutrient intake using the AME approach, despite a dependency on these assumptions, the AME approach is becoming standard practice.

Differences between estimates of apparent nutrient intake (using HCES data with the AME approach) and nutrient intakes (using individual dietary data) reported in the literature ranged between 12% and 72%, and were greater for young children based on results by Karageorgou et al. ^(19)^. Poor agreement for young children was likely influenced by the inability of HCES to capture the nutrient contributions of breastmilk and the distribution of nutrients from foods consumed only by young children to the entire household (e.g. infant formula milk, feeding cereals); none of the publications identified by this review made adjustments for these nutrient contributions for young children. Both household- and individual-level recall are subject to measurement error, but the size of the difference for young children compared with other age groups suggests that HCES food consumption data are often not particularly accurate for young children. Using HCES may lead to systematic overestimation of nutrient intakes for infants and young children when adjustments are not made for breast milk intakes and subsequent estimates of apparent micronutrient intake and inadequacy should be interpreted with caution. These differences, coupled with the increasing availability and use of HCES data for describing and assessing the micronutrient adequacy of diets across populations, suggest more research is necessary to inform the development of a structured framework to guide the use and nuanced interpretation of HCES nutrient supply data that reflect its limitations.

The use of HCESs for nutrient analysis has a number of strengths. The HCES food consumption module is considerably less laborious and less expensive to conduct than national scale individual-level dietary surveys^(42)^. HCESs are conducted regularly and data are typically made publicly available. HCESs are typically based on 7- to 30-day recall periods which pose some advantages compared to a single 24-hour dietary recall estimates when examining associations between food consumption and determinants affecting diets as HCESs account for day-to-day variation that may attenuate associations ^(43)^. In addition, large-scale individual-level dietary intake data are not routinely collected or available in many settings, meaning that the alternative to HCES are national-level Food Balance Sheets^(11)^, which, among other limitations, lack sub-national resolution.

While HCES data can be used to estimate household-level nutrient supply, HCES data do not provide information about the distribution of foods consumed among household members. A majority of studies used the AME approach to convert from household-level to individual-level estimates, however the following points are important to consider. First, the literature demonstrated differences between nutrient intake/apparent intake estimated using individual-level dietary data versus HCES with the AME approach, potentially driven by numerous factors including food consumption patterns ^(19,33,35)^ and differences in diet between sex and age groups ^(37,44)^. This presents challenges when conducting individual-level sub-group analyses using HCES data and isolating certain sub-groups for analysis may pose problems when using HCES data, considering food consumption is reported at the household level by one member, generally the head female. Foods regularly consumed away from home by other family members may be systematically under-reported, although the collection of household food supply data is evolving to adjust for foods consumed away from home, especially in urban contexts^(13,24)^. Second, it is unknown how individual model parameters used in the AME approach, such as physical activity level and body weights, might affect apparent nutrient intake estimates, as few publications reported sensitivity analyses evaluating this. Additional research exploring the sensitivity of AME factors to individual parameter assumptions would improve general understanding of how much these broad population assumptions impact inadequacy estimates. Third, HCES data quality can vary between countries due to differences in HCES questionnaire design, leading to varying availability of important demographic variables, such as pregnancy status. These data are important to characterize household member nutrient requirements and the lack of these data increases uncertainty in estimates of apparent intake and inadequacy.

Uncertainty in how food is allocated within households and if intrahousehold allocation factors can be generalized across different contexts remains a key limitation in the use of HCES data to estimate individual-level apparent nutrient intake. Evidence from Bangladesh suggests that household heads (generally adult males) received proportionally more dietary energy compared to their spouses and children than equitable intra-household food allocation using the AME approach would suggest ^(36)^. Yet contrasting results were found in Ethiopia, where groups hypothesized to be more “vulnerable” (i.e. women and children) were found using 24-hour dietary recall data to consume a greater share of energy and protein in relation to their dietary requirements than their male and adult counterparts (although children under 5 years old were excluded from the regression analysis) ^(45)^. While differences in these results may be due to cultural differences between countries, understanding general uncertainty in intrahousehold allocation of food is important for subgroups who are often the focus of nutrition policy ^(46)^, such as pregnant or lactating women, women of reproductive age, infants, and children. Due to this, individual-level surveys (including micronutrient biomarker assessments and individual dietary assessments) will remain important when characterizing the micronutrient status of these sub-groups. HCES data do, however, serve as a potential resource to identify dietary nutrient shortfalls putting populations at risk of deficiency, and this information may guide the design of individual-level surveys. For example, HCES data can be used to highlight regional and socioeconomic variation and seasonal fluctuations in dietary nutrient supply, which can be useful when developing sampling units for future individual-level surveys.

Diets in low- and lower-middle income countries are affected by a myriad of social determinants ^(47)^, which must be addressed by policies intended to improve equity in the broader food system ^(9)^. This review demonstrates that estimating nutrient supply from HCES data may help identify populations at risk of nutrient deficiencies due to poor diets. The literature identified in this review used HCES data to disaggregate nutrient supply results by well-documented social factors affecting diets, such as wealth ^(41,48,49)^, education ^(50,51)^, geography ^(49,52)^, and ruralness ^(41,53,54)^. HCESs collect microdata describing a wide range of socio-economic and geographic determinants of diets, which provides the opportunity to explore the mechanisms driving these associations in great depth. Considering the original intention of HCESs was to provide microdata to characterize poverty and social welfare in low- and lower-middle income countries, there exist a number of potential opportunities to combine other variables already collected in these surveys with nutrition metrics to identify vulnerable populations and integrate this information into the development of nutrition policies that promote equity. Additionally, characterizing these high-risk groups from information provided by HCESs can help guide nutrition interventions (e.g. do high-risk populations routinely purchase staple foods or food vehicles suitable for fortification?). To better address equity in nutrition policy, there lies potential opportunities for using HCES data to identify and target vulnerable populations disproportionally affected by key socioeconomic and geographic determinants affecting diets. Further research is required to identify indicators of social determinants of poor-quality diets from HCESs and their relation to the specific mechanisms that affect nutrient supply, to inform consistent applications to designing and monitoring nutrition programs and policy.

The growing evidence-base guiding the use of HCES data to estimate nutrient supply has highlighted the potential opportunities HCES data have in informing food systems interventions. As this practice becomes more common, consistency in processing HCES data and reporting results will be important for comparability between findings. While estimating nutrient supply from HCES data will always require time and effort, detailed standard operating procedures, repositories for processed data and descriptive methods, access to standard weight/measure conversions, and open-sourced data processing scripts could encourage further analyses, improve consistency between studies, and facilitate research collaboration. In addition, to further understand what insights and to what degree of certainty HCES dietary analyses can contribute to understanding the adequacy of diets across populations, more research is needed to compare HCES-estimates of nutrient adequacy derived from nutrient density compared to the AME approach, as well as to adequacy estimates from individual 24-hour dietary recall data in various country contexts.

This systematic review had a number of strengths. The screening procedure was exhaustive, including a broad database search and snowballing. This resulted in a wide range of publications from peer-reviewed journals, working papers from multi-national institutions and other grey literature. Our study also extracted data describing the process of transforming HCES data to estimate nutrient supply and social determinants that may affect diets. This study, however, had limitations. There were very few publications that applied the nutrient density approach, making it difficult to draw conclusions regarding potential variation in assumptions and applications of the metric. In addition, while the screening of results from the database search was independently undertaken by two individuals, review of identified publications during screening was only conducted by one.

Estimates of nutrient supply and dietary quality using HCES data can play an important role in micronutrient surveillance and the design of interventions to tackle micronutrient deficiencies. However, HCES remain under-exploited for the identification of vulnerable populations at greatest risk of micronutrient deficiencies and for the design of effective and equitable micronutrient interventions. Further research is required to understand the implications of key methodological decisions when building models using HCES data in an effort to inform national nutrition policy.

# REFERENCES

1. UNICEF, The World Bank, US Agency for International Development, Global Alliance for Improved Nutrition, Micronutrient Initiative FFI (2009) *Investing in the Future: A United Call to Action on Vitamin and Mineral Deficiencies*. Ottawa.

2. Black RE, Victora CG, Walker SP, et al. (2013) Maternal and child undernutrition and overweight in low-income and middle-income countries. *Lancet* **382**, 427–451.

3. Dary O & Imhoff-Kunsch B (2012) Measurement of food consumption to inform food fortification and other nutrition programs: an introduction to methods and their application. *Food Nutr. Bull.* **33**, S141–S145.

4. Ford ND, Bichha RP, Parajuli KR, et al. (2019) Age, Ethnicity, Glucose-6-Phosphate Dehydrogenase Deficiency, Micronutrient Powder Intake, and Biomarkers of Micronutrient Status, Infection, and Inflammation Are Associated with Anemia Among Children 6–59 Months in Nepal. *J. Nutr.* **150**, 929–937.

5. Ford ND, Bichha RP, Parajuli KR, et al. (2020) Factors associated with anaemia in a nationally representative sample of nonpregnant women of reproductive age in Nepal. *Matern. Child Nutr.* **e12953**.

6. Bailey RL, West KP & Black RE (2015) The epidemiology of global micronutrient deficiencies. *Ann. Nutr. Metab.* **66**, 22–33.

7. Singh MB, Fotedar R & Lakshminarayana J (2009) Micronutrient deficiency status among women of desert areas of western Rajasthan, India. *Public Health Nutr.* **12**, 624–9.

8. Kulwa KBM, Verstraeten R, Bouckaert KP, et al. (2014) Effectiveness of a nutrition education package in improving feeding practices, dietary adequacy and growth of infants and young children in rural Tanzania: Rationale, design and methods of a cluster randomised trial. *BMC Public Health* **14**, 1077.

9. Independent Expert Group of the Global Nutrition Report (2020) *2020 Global Nutrition Report: Act on equity to end malnutrition*. Bristol.

10. Rhodes EC, Hennink M, Jefferds MED, et al. (2019) Integrating micronutrient status assessment into the 2015–2016 Malawi Demographic and Health Survey: A qualitative evaluation. *Matern. Child Nutr.* **15 Suppl 1**, e12734.

11. Beal T, Massiot E, Arsenault JE, et al. (2017) Global trends in dietary micronutrient supplies and estimated prevalence of inadequate intakes. *PLoS One* **12**, e0175554.

12. Gibson RS, Ruth Charrondiere U & Bell W (2017) Measurement errors in dietary assessment using self-reported 24-hour recalls in low-income countries and strategies for their prevention. *Adv. Nutr.* **8**, 980–991.

13. Zezza A, Carletto C, Fiedler JL, et al. (2017) Food counts. Measuring food consumption and expenditures in household consumption and expenditure surveys (HCES). Introduction to the special issue. *Food Policy* **72**, 1–6.

14. Food & Agriculture Organization of the United Nations & The World Bank Group (2018) *Food data collection in household consumption and expenditure surveys*. Rome.

15. Grosh M & Glewwe P (2000) *Designing household survey questionnaires for developing countries: lessons from 15 years of the living standards measurement study*. Washington, D.C..

16. Carletto C & Gourlay S (2019) A thing of the past? Household surveys in a rapidly evolving (agricultural) data landscape: Insights from the LSMS-ISA. *Agric. Econ.* **50**, 51–62.

17. Coates J, Colaiezzi B, Fiedler JL, et al. (2012) A program needs-driven approach to selecting dietary assessment methods for decision-making in food fortification programs. *Food Nutr. Bull.* **33**, S146-56.

18. Fiedler JL, Smitz MF, Dupriez O, et al. (2008) Household income and expenditure surveys: A tool for accelerating the development of evidence-based fortification programs. *Food Nutr. Bull.*

19. Karageorgou D, Imamura F, Zhang J, et al. (2018) Assessing dietary intakes from household budget surveys: A national analysis in Bangladesh. *PLoS One* **13**, e0202831.

20. Murphy S, Ruel M & Carriquiry A (2012) Should Household Consumption and Expenditures Surveys (HCES) be used for nutritional assessment and planning? *Food Nutr. Bull.* **33**, S235-41.

21. Fiedler JL, Lividini K, Bermudez OI, et al. (2012) Household Consumption and Expenditures Surveys (HCES): a primer for food and nutrition analysts in low- and middle-income countries. *Food Nutr. Bull.* **33**, S170-84.

22. Food and Agriculture Organization of the United Nations & The World Bank (2018) *Food data collection in household consumption and expenditure surveys: Guidelines for low- and middle-income countries*. Rome.

23. Engle-Stone R, Sununtnasuk C & Fiedler JL (2017) Investigating the significance of the data collection period of household consumption and expenditures surveys for food and nutrition policymaking: Analysis of the 2010 Bangladesh household income and expenditure survey. *Food Policy* **72**, 72–80.

24. Fiedler JL & Yadav S (2017) How can we better capture food away from Home? Lessons from India’s linking person-level meal and household-level food data. *Food Policy* **72**, 81–93.

25. Conforti P, Grünberger K & Troubat N (2017) The impact of survey characteristics on the measurement of food consumption. *Food Policy* **72**, 43–52.

26. Bell W, Coates JC, Rogers BL, et al. (2019) Getting the food list ‘right’: An approach for the development of nutrition-relevant food lists for household consumption and expenditure surveys. *Public Health Nutr.* **22**, 246–256.

27. Moher D, Liberati A, Tetzlaff J, et al. (2009) Preferred Reporting Items for Systematic Reviews and Meta-Analyses: The PRISMA Statement. *PLoS Med.* **6**, e1000097.

28. Badampudi D, Wohlin C & Petersen K (2015) *Experiences from using snowballing and database searches in systematic literature studies*. *ACM Int. Conf. Proceeding Ser.*, vol. 17.

29. The World Bank (2019) World Bank Country Classifications. Washington, D.C.; https://datahelpdesk.worldbank.org/knowledgebase/articles/906519-world-bank-country-and-lending-groups.

30. O’Neill J, Tabish H, Welch V, et al. (2014) Applying an equity lens to interventions: Using PROGRESS ensures consideration of socially stratifying factors to illuminate inequities in health. *J. Clin. Epidemiol.* **67**, 56–64.

31. Weisell R & Dop MC (2012) The adult male equivalent concept and its application to Household Consumption and Expenditures Surveys (HCES). *Food Nutr. Bull.* **33**, S157-62.

32. Vossenaar M, Solomons N, Monterrosa E, et al. (2018) Nutrient Density as a Dimension of Dietary Quality density approach in a multicenter evaluation. *Sight Life* **32**, 172–176.

33. Bromage S, Rosner B, Rich-Edwards JW, et al. (2018) Comparison of methods for estimating dietary food and nutrient intakes and intake densities from household consumption and expenditure data in Mongolia. *Nutrients* **10**, 703.

34. Engle-Stone R & Brown KH (2015) Comparison of a household consumption and expenditures survey with nationally representative food frequency questionnaire and 24-hour dietary recall data for assessing consumption of fortifiable foods by women and young children in Cameroon. *Food Nutr. Bull.* **36**, 211–30.

35. Dary O & Jariseta ZR (2012) Validation of dietary applications of Household Consumption and Expenditures Surveys (HCES) against a 24-hour recall method in Uganda. *Food Nutr. Bull.* **33**, S190-8.

36. D’Souza A & Tandon S (2015) *Using Household and Intrahousehold Data To Assess Food Insecurity: Evidence From Bangladesh*. *ERR-190*. Washington, D.C..

37. Lividini K, Fiedler JL & Bermudez OI (2013) Policy implications of using a Household Consumption and Expenditures Survey versus an Observed-Weighed Food Record Survey to design a food fortification program. *Food Nutr. Bull.* **34**, 520–32.

38. Jariseta ZR, Dary O, Fiedler JL, et al. (2012) Comparison of estimates of the nutrient density of the diet of women and children in Uganda by Household Consumption and Expenditures Surveys (HCES) and 24-hour recall. *Food Nutr. Bull.* **33**, S199-207.

39. Howe LD, Galobardes B, Matijasevich A, et al. (2012) Measuring socio-economic position for epidemiological studies in low-and middle-income countries: A methods of measurement in epidemiology paper. *Int. J. Epidemiol.* **41**, 871–886.

40. Russell J, Lechner A, Hanich Q, et al. (2018) Assessing food security using household consumption expenditure surveys (HCES): A scoping literature review. *Public Health Nutr.* **21**, 2200–2210.

41. Gilbert R, Benson T & Ecker O (2019) *Are Malawian diets changing? An assessment of nutrient consumption and dietary patterns using household-level evidence from 2010/11 and 2016/17*. *MaSSP Work. Pap. 30*. Washington, DC.

42. Fiedler JL, Martin-Prével Y & Moursi M (2013) Relative costs of 24-hour recall and Household Consumption and Expenditures Surveys for nutrition analysis. *Food Nutr. Bull.* **34**, 318–30.

43. Food & Agriculture Organization of the United Nations (2018) *Dietary Assessment: A resource guide to method selection and application in low resource settings.* Rome: .

44. Sununtnasuk C & Fiedler JL (2017) Can household-based food consumption surveys be used to make inferences about nutrient intakes and inadequacies? A Bangladesh case study. *Food Policy* **72**, 121–131.

45. Coates J, Patenaude BN, Rogers BL, et al. (2018) Intra-household nutrient inequity in rural Ethiopia. *Food Policy* **81**, 82–94.

46. Schwarzenberg SJ & Georgieff MK (2018) Advocacy for improving nutrition in the first 1000 days to support childhood development and adult health. *Pediatrics* **141**, e20173716.

47. Harris-Fry H, Shrestha N, Costello A, et al. (2017) Determinants of intra-household food allocation between adults in South Asia - A systematic review. *Int. J. Equity Health* **16**, 1–21.

48. Hjelm L, Mathiassen A & Wadhwa A (2016) Measuring Poverty for Food Security Analysis: Consumption-Versus Asset-Based Approaches. *Food Nutr. Bull.* **37**, 275–289.

49. Joy EJM, Kumssa DB, Broadley MR, et al. (2015) Dietary mineral supplies in Malawi: spatial and socioeconomic assessment. *BMC Nutr.* **1**, 42.

50. Trinh Thi H, Simioni M & Thomas-Agnan C (2018) Decomposition of changes in the consumption of macronutrients in Vietnam between 2004 and 2014. *Econ. Hum. Biol.* **31**, 259–275.

51. Nguyen MC & Winters P (2011) The impact of migration on food consumption patterns: The case of Vietnam. *Food Policy* **36**, 71–87.

52. Bermudez OI, Lividini K, Smitz MF, et al. (2012) Estimating micronutrient intakes from Household Consumption and Expenditures Surveys (HCES): an example from Bangladesh. *Food Nutr. Bull.* **33**, S208-13.

53. Mathiassen A & Hollema S (2014) What is the effect of physical activity level on food consumption, energy deficiency, and dietary diversity? *Food Nutr. Bull.* **35**, 351–360.

54. Smith MR, DeFries R, Chhatre A, et al. (2019) Inadequate Zinc Intake in India: Past, Present, and Future. *Food Nutr. Bull.* **40**, 26–40.

55. Gibson RS (2005) Principles of Nutritional Assessment. *Oxford Univ. Press. Inc*.

#

# TABLES.

***Table 1.*** *Definitions of terms*

| **Term** | **Unit level** | **Nutritional assessment type** | **Definition** |
| --- | --- | --- | --- |
| Apparent nutrient intake ^(17,55)^ | Population | Household consumption & expenditure surveys | Nutrient intake of a population estimated by assuming intrahousehold food consumption is based on proportional energy requirements. |
| Apparent nutrient intake inadequacy ^(17,55)^ | Population | Household consumption & expenditure surveys | Estimate to indicate when apparent nutrient intakes are below the estimated average requirement (EAR) for a population group depending on age-, sex-, and specific physiological stage (e.g. pregnancy and lactation). |
| Food consumption ^(55)^ | Individual | Individual dietary assessment | Measure of the average food consumption by an individual during a period of time, usually expressed per day. Necessary to estimate nutrient intake. |
| Household food supply ^(55)^ | Household | Household consumption & expenditure surveys | Self-reported estimate of the total amount of food available for consumption in the household over a fixed time period (usually 7 to 30 days), generally excluding foods consumed away from home. |
| Nutrient deficiency ^(55)^ | Individual/ population | Nutrient biomarker assessments | Biological measure defined by the range where clinical or functional manifestations of nutrient deficiencies present. May be caused by various factors, including inadequate nutrient intake. |
| Nutrient density ^(32)^ | Household | Household consumption & expenditure surveys | An indicator of dietary quality expressed as the ratio of the nutrient supply of the diet to the energy supply provided by the same diet. |
| Nutrient intake ^(55)^ | Individual | Individual dietary assessment | Estimate of the average intake of nutrients, mostly through foods consumed by an individual. Calculated from quantitative data on food consumption and nutrient content compiled in food composition databases or tables. |
| Nutrient intake inadequacy ^(55)^ | Individual/ population | Individual dietary assessment | Estimate to indicate when nutrient intakes are below the recommended nutrient intake (RNI, which is replaced in some countries for Recommended Daily Allowance - RDA) for individuals, or below the estimated average requirement (EAR) for a population group depending on age-, sex-, and specific physiological stage (e.g. pregnancy and lactation). |
| Nutrient supply ^(55)^ | Household | Household consumption & expenditure surveys | Estimate of nutrients available for consumption through foods for the household. Calculated from quantitative data on household food supply and nutrient content compiled in food composition databases or tables. |

***Table 2.*** *Publications comparing estimates of apparent nutrient and energy intake using Household Consumption & Expenditure Survey (HCES) adult male equivalent (AME) method to individual-level dietary assessment data for total populations and child populations.*

| **Reference;**  **country;**  **summary metric** | **Individual dietary assessment data (N)** | **HCES-AME dietary data,**  **(n households)** | **Study sample population (n)** | **Nutrient** | **Individual intake** | **HCES-AME apparent intake** | **Percentage point**  **difference** |
| --- | --- | --- | --- | --- | --- | --- | --- |
| Bromage *et al.* (2018) ^(33)^;  Mongolia;  means | 24H individual recall  (N=4070) | 7 to 30-day recall of household meals  (n=1012) | Total population  (n=4070) | Energy, kcal | 1863 | 2951 | 58 |
|  |  |  |  | Folate, μg | 132 | 208 | 58 |
|  |  |  |  | Iron, mg | 10 | 16 | 60 |
|  |  |  |  | Vitamin A, μg | 448 | 621 | 39 |
|  |  |  |  | Zinc, mg | 11 | 19 | 72 |
| D’Souza & Tandon (2015) ;  Bangladesh;  means | 24H recall of household meals and proportions consumed by individuals  (N=21,795) | 7-day recall of household meals  (n=5319) | Total population  (n=21,795) | Energy, kcal | 2436 | 2718 | 12 |
| Karageorgou *et al.* (2018) ^(19)^;  Bangladesh;  means | 24H recall of household meals and proportions consumed by individuals  (N=22,173) | 7-day recall of household meals  (n=5503) | Total population  (n=22,173) | Energy, kcal | 2065 | 2322 | 12 |
|  |  |  |  | Folate, μg | 121 | 157 | 31 |
|  |  |  |  | Iron, mg | 9.9 | 12 | 21 |
|  |  |  |  | Vitamin A RAE, μg | 214 | 323 | 50 |
|  |  |  |  | Zinc, mg | 8.6 | 9.8 | 14 |
|  |  |  | Children (<5 years)  (n=2807) | Energy, kcal | 880 | 1130 | 28 |
|  |  |  |  | Folate, μg | 55 | 76 | 40 |
|  |  |  |  | Iron, mg | 4.2 | 5.8 | 38 |
|  |  |  |  | Vitamin A RAE, μg | 108 | 158 | 47 |
|  |  |  |  | Zinc, mg | 3.6 | 4.8 | 31 |
| Lividini *et al.* (2013) ^(37)^;  Rajshahi, Bangladesh;  medians | 2 non-consecutive days of 12-hour observed weighed food records & 12-hour dietary recall (n=477) | 14-day recall of  households containing children age 2-3 (n=513) | Children (2-3 years)  (n=237) | Energy, kcal | 905 | 1098 | 18 |
| Lividini *et al.* (2013) ^(37)^;  Dhaka, Bangladesh;  medians | 2 non-consecutive days of 12-hour observed weighed food records & 12-hour dietary recall (n=464) | 14-day recall of  households containing children age 2-3 (n=678) | Children (2-3 years)  (n=226) | Energy, kcal | 868 | 1104 | 21 |
