# Supplementary material for: Systematic review of metrics used to characterise dietary nutrient supply from household consumption and expenditure surveys
Source: Public Health Nutr. 2022 Jan 13;25(5):1153–65. doi: 10.1017/S1368980022000118 (PMC9991734; doi:10.1017/S1368980022000118)
Supplement: Supplementary file 1 [file S1368980022000118sup.zip › S1368980022000118sup003.docx]

**SUPPLEMENTARY MATERIAL**

Table of Contents

[Table S1. Publications reviewed and the metrics reported 2](#_Toc76569015)

[Table S2. Characteristics of Household Consumption & Expenditure Survey (HCES) food consumption data used in the included publications 4](#_Toc76569016)

[Table S3. Publications comparing apparent nutrient or energy intake estimates using HCES adult male equivalent methods to intake estimates calculated using individual-level dietary intake data for total populations (expanded to include all reported nutrients) 6](#_Toc76569017)

[Table S4. Publications comparing apparent nutrient, energy or fortifiable food item intake estimates using HCES adult male equivalent methods to intake estimates calculated using individual-level dietary intake data for infants and children where no studies adjusted for assumed or recommended breastmilk intake (expanded to include all reported nutrients and food items) 8](#_Toc76569018)

[Table S5. Publications comparing apparent nutrient, energy or fortifiable food item intake estimates using HCES adult male equivalent methods to intake estimates calculated using individual-level dietary intake data for women of reproductive age (WRA) 11](#_Toc76569019)

[Appendix 1. Original protocol of systematic review developed according to PRISMA guidelines 13](#_Toc76569020)

[Appendix 2. Search algorithms returning publications in database search 13](#_Toc76569021)

[Appendix 3. Information extracted from the included literature 17](#_Toc76569022)

# Table S1. Publications reviewed and the metrics reported

| **Ref #** | **Reference** | **Country** | **Household consumption & expenditure data source** | **Supply/**  **per capita /day** | **Supply/ AME/day** | | **Nutrient density** | **Estimated inadequacy** |
| --- | --- | --- | --- | --- | --- | --- | --- | --- |
| ^(1)^ | Ambagna & Dury (2016) | Cameroon | Deuxième/Troisième Enquête Camerounaise auprès des Ménages |  | | X |  | X |
| ^(2)^ | Beegle et al. (2010) | Tanzania | Survey modelled after Household Budget Survey (2006/07) | X | |  |  |  |
| ^(3)^ | Bell et al. (2019) | Bangladesh | Bangladesh Integrated Household Survey (2011/12) |  | |  |  |  |
| ^(4)^ | Bermudez et al. (2012) | Bangladesh | ﻿Bangladesh Household Income and Expenditure Survey (2005) |  | | X |  | X |
| ^(5)^ | Bogard & Mamun (2016) | Bangladesh | Bangladesh Integrated Household Survey (2011/12) |  | |  |  |  |
| ^(6)^ | Bogard et al. (2017) | Bangladesh | Household Income and Expenditure Survey (1991, 2000, 2010) |  | | X |  |  |
| ^(7)^ | Bromage et al. (2018) | Mongolia | Mongolian Household Socio-Economic Survey (2012, 2014) | X | | X | X |  |
| ^(8)^ | Chakrabarti et al. (2018) | India | ﻿61^st^/68^th^ round of the ﻿National Sample Survey (2004/05, 2011/12) |  | |  |  |  |
| ^(9)^ | Chege et al. (2015) | Kenya | Survey modelled after standard HCES food consumption module |  | | X |  | X |
| ^(10)^ | D’Souza & Tandon (2019) | Bangladesh | Bangladesh Integrated Household Survey (2011/12) |  | | X |  | X |
| ^(11)^ | Dary & Jariseta (2012) | Uganda | National Household Survey (2005/06) |  | | X |  |  |
| ^(12)^ | de Weerdt et al. (2014) | Tanzania | Survey modelled after Household Budget Survey (2006/07) | X | |  |  | X |
| ^(13)^ | DeFries et al. (2018) | India | 7 rounds of the National Sample Survey | X | |  |  | X |
| ^(14)^ | Donovan & Massingue (2007) | Mozambique | Trabalho de Inquérito Agricola (2002, 2005) |  | | X |  |  |
| ^(15)^ | Dop et al. (2012) | Cape Verde | I﻿nquérito às Despesas e Receitas Famílias (2001/02) |  | | X | X |  |
| ^(16)^ | Ecker & Qaim (2011) | Malawi | Second Integrated Household Survey (2004/05) | X | |  |  | X |
| ^(17)^ | Ecker & Qaim (2008) | Malawi | Second Integrated Household Survey (2004/05) | X | |  |  | X |
| ^(18)^ | Engle-Stone & Brown (2015) | Cameroon | Troisième Enquête Camerounaise auprès des Ménages |  | | X |  |  |
| ^(19)^ | Engle-Stone et al. (2017) | Bangladesh | ﻿Bangladesh Household Income and Expenditure Survey (2010) |  | | X |  | X |
| ^(20)^ | Euler et al. (2017) | Indonesia | Survey modelled after Household Socio-Economic Survey |  | | X |  | X |
| ^(21)^ | Fiedler (2014) | Bangladesh | ﻿Bangladesh Household Income and Expenditure Survey (2010) |  | | X | X | X |
| ^(22)^ | Fiedler et al. (2012) | India | ﻿61st round of the ﻿National Sample Survey (2004/05) |  | | X |  | X |
| ^(23)^ | Fiedler & Lividini (2014) | Zambia | Living Conditions Monitoring Survey V (2006) |  | | X |  | X |
| ^(24)^ | Fiedler et al. (2015) | Bangladesh | ﻿Bangladesh Household Income and Expenditure Survey (2010) |  | | X |  | X |
| ^(25)^ | Fiedler et al. (2016) | Bangladesh | Bangladesh Household Income and Expenditure Survey (2005) |  | | X |  | X |
| ^(26)^ | Fiedler et al. (2015) | Bangladesh | Bangladesh Household Income and Expenditure Survey (2005) |  | | X |  | X |
| ^(26)^ | Fiedler et al. (2013) | Zambia | Living Conditions Monitoring Survey V (2006) |  | | X |  | X |
| ^(27)^ | Fiedler et al. (2013) | Zambia | Living Conditions Monitoring Survey V (2006) |  | | X |  | X |
| ^(28)^ | Gilbert et al. (2019) | Malawi | Third/Fourth Integrated Household Survey (2010/11, 2016/17) | X | |  |  | X |
| ^(29)^ | Hall et al. (2019) | Malawi | Third Integrated Household Survey (2010/11) |  | | X |  | X |
| ^(30)^ | Hirvonen et al. (2016) | Ethiopia | ﻿Household Consumption Expenditure Survey (2010/11) | X | |  |  |  |
| ^(31)^ | Hjelm et al. (2016) | Madagascar, Malawi, Nepal, Tanzania, Uganda | Enquête Nationale Sur le Suivi des Objectifs du Millénaire pour le Développement à Madagascar (ENSOMD) ;  Third Integrated Household Survey (2010/11);  Nepal Living Standard Survey (2010/11);  Tanzania National Panel Survey (2010/2011);  Uganda National Panel Survey (2009/2010) | X | | X |  | X |

| **Ref #** | **Reference** | **Country** | **Household consumption & expenditure data source** | **Supply/ per capita/ day** | **Supply/ AME/day** | **Nutrient density** | **Estimated**  **inadequacy** |
| --- | --- | --- | --- | --- | --- | --- | --- |
| ^(32)^ | Imhoff-Kunsch et al. (2019) | Solomon Islands | ﻿Household Income and Expenditure Survey (2012/13) |  | X |  | X |
| ^(33)^ | Jarista et al. (2012) | Uganda | National Household Survey (2005/06) |  | X | X | X |
| ^(34)^ | Jati et al. (2012) | Indonesia | ﻿Household Socio-Economic Survey (BPS 2008) |  |  | X | X |
| ^(35)^ | Johnecheck & Holland (2007) | Afghanistan | National Risk and Vulnerability Assessment (2003) | X |  |  | X |
| ^(36)^ | Joy et al. (2015) | Malawi | Third Integrated Household Survey (2010/11) |  | X |  | X |
| ^(37)^ | Kankwamba & Kornher (2019) | Malawi | Third/Fourth Integrated Household Survey (2010/11, 2016/17) | X |  |  |  |
| ^(38)^ | Karageorgou et al. (2018) | Bangladesh | Bangladesh Integrated Household Survey (2011/12) | X | X |  |  |
| ^(39)^ | Lividini & Fiedler (2015) | Zambia | Living Conditions Monitoring Survey V (2006) |  | X |  | X |
| ^(40)^ | Lividini et al. (2013) | Bangladesh | Bangladesh Household Income and Expenditure Survey (2005) |  | X |  | X |
| ^(41)^ | Marivoet et al. (2018) | Dem. Rep. of the Congo | ﻿﻿Enquête 123: données transversales sur la consommation des ménages collectées |  | X |  | X |
| ^(42)^ | Mathiassen & Hollema (2014) | Nepal, Uganda | ﻿Uganda National Panel Survey (2009/10);  ﻿Nepal Living Standard Survey (2010/11) |  |  |  | X |
| ^(43)^ | Mishra & Ray (2009) | Vietnam | Vietnamese Household Living Standard Surveys (1993, 1998, 2004) | X |  |  | X |
| ^(44)^ | Molini (2006) | Vietnam | Vietnamese Household Living Standard Survey (1993, 1998) | X |  |  |  |
| ^(45)^ | Mwangi et al. (2016) | Bangladesh | Bangladesh Integrated Household Survey (2011/12) |  | X |  | X |
| ^(46)^ | Nguyen & Winters (2011) | Vietnam | ﻿Vietnam Household Living Standards Surveys | X |  |  |  |
| ^(47)^ | Rahman (2012) | Bangladesh | Bangladesh Household Income and Expenditure Survey (2005) | X |  |  |  |
| ^(48)^ | Rose & Charlton (2002) | South Africa | ﻿Income and Expenditure Survey (1995) |  | X |  | X |
| ^(49)^ | Skoufias (2002) | Indonesia | National Socio-Economic Surveys (1996, 1999) | X |  |  |  |
| ^(50)^ | Smith et al. (2006) | Burundi, Ethiopia, Ghana, Guinea, Kenya, Malawi, Mozambique, Rwanda, Senegal, Tanzania, Uganda, Zambia | Étude nationale sur les conditions de vie des populations (1998)  Household Income, Consumption & Expenditure Survey (1999/2000)  Ghana Living Standards Survey 4  Enquête intégrale sur les conditions de vie des ménages guinéen  Kenya Welfare Monitoring Survey III  Malawi Integrated Household Survey (19997/98)  Mozambique Inqerito nacional aos agregados familiareas sobre as condicoes de vida  Enquête intégrale sur les conditions de vie des ménages rwandaise  Enquête Sénégalaise auprès des ménages II  Tanzanian Household Budget Survey  Ugandan National Household Survey (1999/2000)  Zambia Living Conditions Monitoring Survey I (1996) | X |  |  | X |
| ^(51)^ | Smith et al. (2019) | India | 7 rounds of the National Sample Survey | X |  |  | X |
| ^(52)^ | Tandon & Landes (2010) | India | ﻿61st round of the ﻿National Sample Survey (2004/05) | X |  |  |  |
| ^(53)^ | Trinh Thi et al. (2018) | Vietnam | Vietnam Household Living Standards Survey | X |  |  |  |
| ^(54)^ | Troubat & Grünberger (2017) | Mongolia | Mongolian Household Socio-Economic Survey (2007/08) | X |  |  |  |
| ^(55)^ | Ulimwengu et al. (2011) | Uganda | National Household Survey (2005/06) | X |  |  | X |
| ^(56)^ | Verduzco-Gallo et al. (2014) | Malawi | Second/Third Integrated Household Survey (2010/11, 2016/17) | X |  |  | X |
| ^(57)^ | Weinberger (2004) | India | ﻿50^th^ round of the ﻿National Sample Survey (1993/94) |  | X |  | X |
| ^(58)^ | Worku et al. (2017) | Ethiopia | ﻿Household Consumption Expenditure Surveys |  | X |  |  |

# Table S2. Characteristics of Household Consumption & Expenditure Survey (HCES) food consumption data used in the included publications

| **Ref #** | **Reference** | **Country** | **HCES: Type of dietary data** | **HCES: Time horizon** | **Compared HCES to individual intake data?** | **Individual intake: type of data** | **Individual intake: comparability to HCES** | **Individual intake: demographics of individuals** |
| --- | --- | --- | --- | --- | --- | --- | --- | --- |
| ^(1)^ | Ambagna & Dury (2016) | Cameroon | Consumption recall | 10 days (rural) & 15 days (urban) |  |  |  |  |
| ^(2)^ | Beegle et al. (2010) | Tanzania | Consumption recall | 7 days, 14 days, & 12 months |  |  |  |  |
| ^(3)^ | Bell et al. (2019) | Bangladesh | Consumption recall | 7 days |  |  |  |  |
| ^(4)^ | Bermudez et al. (2012) | Bangladesh | Acquisition recall | 14 days |  |  |  |  |
| ^(5)^ | Bogard & Mamun (2016) | Bangladesh | Consumption recall | 7 days |  |  |  |  |
| ^(6)^ | Bogard et al. (2017) | Bangladesh | Consumption recall | 7 days |  |  |  |  |
| ^(7)^ | Bromage et al. (2018) | Mongolia | Consumption recall | 7 days (rural) & 10 to 30 days (urban) | X | Multipass 24-hour individual recall | Individuals of households from HCES sample | All household members |
| ^(8)^ | Chakrabarti et al. (2018) | India | Consumption recall | 1 month |  |  |  |  |
| ^(9)^ | Chege et al. (2015) | Kenya | Consumption recall | 7 days |  |  |  |  |
| ^(10)^ | D’Souza & Tandon (2019) | Bangladesh | Consumption recall | 7 days |  |  |  |  |
| ^(11)^ | Dary & Jariseta (2012) | Uganda | Consumption recall | 7 days | X | Multipass 24-hour individual recall | Different sample^1^ | Children <5 years old & women |
| ^(12)^ | de Weerdt et al. (2014) | Tanzania | Consumption recall | 7 days, 14 days, & 12 months |  |  |  |  |
| ^(13)^ | DeFries et al. (2018) | India | Consumption & expenditure recall | 1 month |  |  |  |  |
| ^(14)^ | Donovan & Massingue (2007) | Mozambique | Acquisition recall | 1 year |  |  |  |  |
| ^(15)^ | Dop et al. (2012) | Cape Verde | Direct weighed observation | 7 days |  |  |  |  |
| ^(16)^ | Ecker & Qaim (2011) | Malawi | Consumption recall | 7 days |  |  |  |  |
| ^(17)^ | Ecker & Qaim (2008) | Malawi | Consumption recall | 7 days |  |  |  |  |
| ^(18)^ | Engle-Stone & Brown (2015) | Cameroon | Acquisition recall | 3 & 7 days | X | Single-pass 24-hour individual recall^2^ | Different sample^1^ | Children <5 years old & women |
| ^(19)^ | Engle-Stone et al. (2017) | Bangladesh | Consumption recall | 2 days |  |  |  |  |
| ^(20)^ | Euler et al. (2017) | Indonesia | Consumption recall | 7 days |  |  |  |  |
| ^(21)^ | Fiedler (2014) | Bangladesh | Consumption recall | 2 days |  |  |  |  |
| ^(22)^ | Fiedler et al. (2012) | India | Consumption recall | 1 month |  |  |  |  |
| ^(23)^ | Fiedler & Lividini (2014) | Zambia | Acquisition recall | 14 days |  |  |  |  |
| ^(24)^ | Fiedler et al. (2015) | Bangladesh | Acquisition recall | 14 days |  |  |  |  |
| ^(25)^ | Fiedler et al. (2016) | Bangladesh | Consumption recall | 2 days |  |  |  |  |
| ^(26)^ | Fiedler et al. (2015) | Bangladesh | Consumption recall | 2 days |  |  |  |  |
| ^(26)^ | Fiedler et al. (2013) | Zambia | Acquisition recall | 14 days |  |  |  |  |
| ^(27)^ | Fiedler et al. (2013) | Zambia | Acquisition recall | 14 days |  |  |  |  |
| ^(28)^ | Gilbert et al. (2019) | Malawi | Consumption recall | 7 days |  |  |  |  |
| ^(29)^ | Hall et al. (2019) | Malawi | Consumption recall | 7 days |  |  |  |  |
| ^(30)^ | Hirvonen et al. (2016) | Ethiopia | Consumption recall | 3 & 4 days |  |  |  |  |
| ^(31)^ | Hjelm et al. (2016) | Madagascar, Malawi, Nepal, Tanzania, Uganda | Acquisition recall | Varies |  |  |  |  |

^1^ Individual dietary data surveyed from a different sample of household compared to HCES sample

^2^ 10% of individuals in the total sample were randomly selected for multipass dietary recall methods

| **Ref #** | **Reference** | **Country** | **HCES: Type of dietary data** | **HCES: Time horizon** | **Compared to individual intake data?** | **Individual intake: type of data** | **Individual intake: comparability to HCES** | **Individual intake: demographics of individuals** |
| --- | --- | --- | --- | --- | --- | --- | --- | --- |
| ^(32)^ | Imhoff-Kunsch et al. (2019) | Solomon Islands | Expenditure diary | 14 days |  |  |  |  |
| ^(33)^ | Jarista et al. (2012) | Uganda | Consumption recall | 7 days |  |  |  |  |
| ^(34)^ | Jati et al. (2012) | Indonesia | Expenditure recall | 7 days |  |  |  |  |
| ^(35)^ | Johnecheck & Holland (2007) | Afghanistan | Consumption recall | 7 days |  |  |  |  |
| ^(36)^ | Joy et al. (2015) | Malawi | Consumption recall | 7 days |  |  |  |  |
| ^(37)^ | Kankwamba & Kornher (2019) | Malawi | Consumption recall | 7 days |  |  |  |  |
| ^(38)^ | Karageorgou et al. (2018) | Bangladesh | Consumption recall | 7 days | X | Single-pass 24-hour individual recall | Individuals of households from HCES sample | All household members |
| ^(39)^ | Lividini & Fiedler (2015) | Zambia | Acquisition recall | 14 days |  |  |  |  |
| ^(40)^ | Lividini et al. (2013) | Bangladesh | Consumption recall | 14 days | X | 2-day observed-weighed food records | Individuals of households from HCES sample | Children <5 years old & women |
| ^(41)^ | Marivoet et al. (2018) | Dem. Rep. of the Congo | Acquisition & expenditure recall | 15 days & 6 to 12 months |  |  |  |  |
| ^(42)^ | Mathiassen & Hollema (2014) | Nepal, Uganda | Acquisition recall | 7 days |  |  |  |  |
| ^(43)^ | Mishra & Ray (2009) | Vietnam | Expenditure recall | 1 year |  |  |  |  |
| ^(44)^ | Molini (2006) | Vietnam | Expenditure recall | 1 year |  |  |  |  |
| ^(45)^ | Mwangi et al. (2016) | Bangladesh | Consumption recall | 7 days |  |  |  |  |
| ^(46)^ | Nguyen & Winters (2011) | Vietnam | Expenditure recall | 1 year |  |  |  |  |
| ^(47)^ | Rahman (2012) | Bangladesh | Consumption recall | 14 days |  |  |  |  |
| ^(48)^ | Rose & Charlton (2002) | South Africa | Expenditure recall | 1 month |  |  |  |  |
| ^(49)^ | Skoufias (2002) | Indonesia | Consumption recall | 7 days |  |  |  |  |
| ^(50)^ | Smith et al. (2006) | Burundi, Ethiopia, Ghana, Guinea, Kenya, Malawi, Mozambique, Rwanda, Senegal, Tanzania, Uganda, Zambia | Acquisition & expenditure recall | Varies |  |  |  |  |
| ^(51)^ | Smith et al. (2019) | India | Expenditure & consumption recall | 1 month |  |  |  |  |
| ^(52)^ | Tandon & Landes (2010) | India | Expenditure recall | 1 month |  |  |  |  |
| ^(53)^ | Trinh Thi et al. (2018) | Vietnam | Expenditure recall | 1 year |  |  |  |  |
| ^(54)^ | Troubat & Grünberger (2017) | Mongolia | Consumption recall | 10 days |  |  |  |  |
| ^(55)^ | Ulimwengu et al. (2011) | Uganda | Consumption recall | 7 days |  |  |  |  |
| ^(56)^ | Verduzco-Gallo et al. (2014) | Malawi | Consumption recall | 7 days |  |  |  |  |
| ^(57)^ | Weinberger (2004) | India | Expenditure recall | 30 days |  |  |  |  |
| ^(58)^ | Worku et al. (2017) | Ethiopia | Consumption recall | 7 days |  |  |  |  |

# **Table S3.** Publications comparing apparent nutrient or energy intake estimates using HCES adult male equivalent methods to intake estimates calculated using individual-level dietary intake data for total populations (expanded to include all reported nutrients)

| **Reference;**  **country;**  **summary metric** | **Individual consumption data (N)** | **HCES dietary data, (n)** | **Study sample population (n)** | **Nutrient** | **Individual estimate** | **HCES AME estimate** | **Percentage point**  **difference** |
| --- | --- | --- | --- | --- | --- | --- | --- |
| ***I. Study compared intake estimates derived from individual-level versus HCES dietary data*** | | | | | | | |
| Bromage *et al.* (2018) ^(7)^;  Mongolia;  means | 24H individual recall  (N=4070) | 7-30 day recall of household meals  (n=1012) | Total population  (n=4070) | Energy, kcal | 1863 | 2951 | 58 |
|  |  |  |  | Carbohydrates, g | 241 | 364 | 51 |
|  |  |  |  | Protein, g | 70 | 120 | 71 |
|  |  |  |  | Total fat, g | 66 | 113 | 71 |
|  |  |  |  | Thiamin, mg | 0.8 | 1.3 | 72 |
|  |  |  |  | Riboflavin, mg | 1.2 | 2.2 | 83 |
|  |  |  |  | Niacin, mg | 13 | 23 | 72 |
|  |  |  |  | Pantothenic acid, mg | 3.1 | 5.5 | 77 |
|  |  |  |  | Vitamin B6, mg | 0.6 | 1.1 | 88 |
|  |  |  |  | Folate, μg | 132 | 208 | 58 |
|  |  |  |  | Vitamin B12, μg | 6.4 | 8.4 | 31 |
|  |  |  |  | Vitamin C, mg | 12 | 33 | 167 |
|  |  |  |  | Vitamin A, μg | 448 | 621 | 39 |
|  |  |  |  | Vitamin D, IU | 26 | 48 | 85 |
|  |  |  |  | Vitamin E, mg | 5.3 | 9.6 | 82 |
|  |  |  |  | Calcium, mg | 432 | 898 | 108 |
|  |  |  |  | Copper, mg | 1.0 | 1.5 | 48 |
|  |  |  |  | Iron, mg | 10 | 16 | 60 |
|  |  |  |  | Magnesium, mg | 168 | 283 | 68 |
|  |  |  |  | Manganese, mg | 2.2 | 3.4 | 55 |
|  |  |  |  | Phosphorous, mg | 907 | 1567 | 73 |
|  |  |  |  | Potassium, mg | 1436 | 2645 | 84 |
|  |  |  |  | Zinc, mg | 11 | 19 | 72 |
| D’Souza & Tandon (2015) ^(10)^;  Bangladesh;  means | 24H recall of household meals and proportions consumed by individuals  (N=21,795) | 7-day recall of household meals  (n=5319) | Total population  (n=21,795) | Energy, kcal | 2436 | 2718 | 12 |
| Karageorgou *et al.* (2018) ^(38)^;  Bangladesh;  means | 24H recall of household meals and proportions consumed by individuals  (N=22,173) | 7-day recall of household meals  (n=5503) | Total population  (n=22,173) | Energy, kcal | 2065 | 2322 | 12 |
|  |  |  |  | Protein, g | 50 | 57 | 13 |
|  |  |  |  | Carbohydrates, g | 398 | 444 | 12 |
|  |  |  |  | Total fat, g | 26 | 30 | 17 |
|  |  |  |  | Vitamin A RAE, μg | 214 | 323 | 50 |
|  |  |  |  | Vitamin D, μg | 1.1 | 1.3 | 18 |
|  |  |  |  | Vitamin E, mg | 4.5 | 5.4 | 20 |
|  |  |  |  | Thiamine, mg | 0.8 | 0.9 | 25 |
|  |  |  |  | Riboflavin, mg | 0.5 | 0.6 | 20 |
|  |  |  |  | Niacin, mg | 14 | 16 | 13 |
|  |  |  |  | Vitamin B6 | 1.2 | 1.4 | 17 |
|  |  |  |  | Folate, μg | 121 | 157 | 31 |
|  |  |  |  | Vitamin C, mg | 42 | 65 | 55 |
|  |  |  |  | Calcium, mg | 274 | 343 | 25 |
|  |  |  |  | Iron, mg | 9.9 | 12 | 21 |
|  |  |  |  | Sodium, mg | 4225 | 5855 | 39 |
|  |  |  |  | Potassium, mg | 1395 | 1745 | 25 |
|  |  |  |  | Magnesium, mg | 321 | 377 | 18 |
|  |  |  |  | Zinc, mg | 8.6 | 9.8 | 14 |

# **Table S4.** Publications comparing apparent nutrient, energy or fortifiable food item intake estimates using HCES adult male equivalent methods to intake estimates calculated using individual-level dietary intake data for infants and children where no studies adjusted for assumed or recommended breastmilk intake (expanded to include all reported nutrients and food items)

| **Reference;**  **country;**  **summary metric** | **Individual dietary assessment data (N)** | **HCES dietary data, (n households)** | **Study sample population (n)** | **Nutrient/ food item** | **Individual intake** | **HCES AME apparent intake** | **Percentage point Difference** |
| --- | --- | --- | --- | --- | --- | --- | --- |
| ***I. Study compared intake estimates derived from individual-level versus HCES dietary data*** | | | | | | | |
| Karageorgou *et al.* (2018) ^(38)^;  Bangladesh;  means | 24H recall of household meals and proportions consumed by individuals  (N=22,173) | 7-day recall of household meals  (n=5503) | Children (<5 years)  (n=2807) | Energy, kcal | 880 | 1130 | 28 |
|  |  |  |  | Protein, g | 22 | 28 | 26 |
|  |  |  |  | Carbohydrates, g | 164 | 216 | 32 |
|  |  |  |  | Total fat, g | 13 | 15 | 10 |
|  |  |  |  | Vitamin A RAE, μg | 108 | 158 | 47 |
|  |  |  |  | Vitamin D, μg | 0.5 | 0.6 | 40 |
|  |  |  |  | Vitamin E, mg | 2.0 | 2.6 | 30 |
|  |  |  |  | Thiamine, mg | 0.3 | 0.4 | 33 |
|  |  |  |  | Riboflavin, mg | 0.3 | 0.3 | 10 |
|  |  |  |  | Niacin, mg | 5.7 | 7.8 | 37 |
|  |  |  |  | Vitamin B6 | 0.5 | 0.7 | 40 |
|  |  |  |  | Folate, μg | 55 | 76 | 40 |
|  |  |  |  | Vitamin C, mg | 18 | 32 | 80 |
|  |  |  |  | Calcium, mg | 151 | 166 | 10 |
|  |  |  |  | Iron, mg | 4.2 | 5.8 | 38 |
|  |  |  |  | Sodium, mg | 1960 | 2887 | 47 |
|  |  |  |  | Potassium, mg | 621 | 850 | 37 |
|  |  |  |  | Magnesium, mg | 132 | 184 | 40 |
|  |  |  |  | Zinc, mg | 3.6 | 4.8 | 31 |
| Lividini *et al.* (2013) ^(40)^;  Rajshahi, Bangladesh;  medians | 2 non-consecutive days of 12-hour observed weighed food records & 12-hour dietary recall (n=477) | 14-day food diary of  households with children age 2-3 (n=513) | Children (2-3 years)  (n=237) | Energy, kcal | 905 | 1098 | 18 |
| Lividini *et al.* (2013) ^(40)^;  Dhaka, Bangladesh;  medians | 2 non-consecutive days of 12-hour observed weighed food records & 12-hour dietary recall (n=464) | 14-day food diary of  households with children age 2-3 (n=678) | Children (2-3 years)  (n=226) | Energy, kcal | 868 | 1104 | 21 |
| ***II. Study compared consumption of fortified food vehicles derived from individual-level versus HCES dietary data*** | | | | | | | |
| Engle-Stone & Brown (2015) ^(18)^;  Cameroon;  medians | 24H individual recall  (N=1794) | Combination of 3 to 7 day recalls and 15-day diary depending on sub-population, different survey sample  (n=4363) | Children (12-59 months) (n=882) | Refined oil, g | 11.7 | 6.2 | -89 |
|  |  |  |  | Wheat flour, g | 49.4 | 24.2 | -104 |
|  |  |  |  | Sugar, g | 19.6 | 7.2 | -172 |
|  |  |  |  | Bouillon, g | 0.9 | 1.3 | 31 |
| Dary & Jariseta (2012) ^(11)^;  Kampala, Uganda;  medians | 24H individual recall | 7-day recall of household meals  (n=314) | Children (2-5 years) | Vegetable oil, g | 4.9 | 7.0 | 30 |
|  |  |  |  | Wheat flour, g | 36.2 | 16.0 | -126 |
|  |  |  |  | Sugar, g | 38.2 | 24.9 | -53 |
|  |  |  |  | Maize flour, g | 49.1 | 24.3 | -102 |
|  |  |  |  | Rice, g | 43.8 | 20.5 | -114 |
| Dary & Jariseta (2012) ^(11)^;  Southwestern Region, Uganda;  medians | 24H individual recall | 7-day recall of household meals  (n=322) | Children (2-5 years) | Vegetable oil, g | 5.7 | 5.0 | -14 |
|  |  |  |  | Wheat flour, g | 32.4 | 7.5 | -332 |
|  |  |  |  | Sugar, g | 23.5 | 11.6 | -103 |
|  |  |  |  | Maize flour, g | 41.8 | 27.0 | -55 |
|  |  |  |  | Rice, g | 33.1 | 18.1 | -83 |
| Dary & Jariseta (2012) ^(11)^;  Northern Region, Uganda;  medians | 24H individual recall | 7-day recall of household meals  (n=321) | Children (2-5 years) | Vegetable oil, g | 9.4 | 3.8 | -147 |
|  |  |  |  | Wheat flour, g | 26.2 | 3.3 | -694 |
|  |  |  |  | Sugar, g | 25.0 | 6.7 | -273 |
|  |  |  |  | Maize flour, g | 69.4 | 10.7 | -549 |
|  |  |  |  | Rice, g | 27.7 | 9.7 | -186 |

# **Table S5.** Publications comparing apparent nutrient, energy or fortifiable food item intake estimates using HCES adult male equivalent methods to intake estimates calculated using individual-level dietary intake data for women of reproductive age (WRA)

| **Reference;**  **country;**  **summary metric** | **Individual dietary assessment data (N)** | **HCES dietary data,**  **(n households)** | **Study sample population (n)** | **Nutrient/ food item** | **Individual intakes** | **HCES AME apparent intakes** | **Percentage point difference** |
| --- | --- | --- | --- | --- | --- | --- | --- |
| ***I. Study compared intake estimates derived from individual-level versus HCES dietary data*** | | | | | | | |
| Karageorgou *et al.* (2018) ^(38)^;  Bangladesh;  means | 24H recall of household meals and proportions consumed by individuals  (N=22,173) | 7 day recall of household meals  (n=5503) | Women  (n=11,671) | Energy, kcal | 1965 | 2180 | 11 |
|  |  |  |  | Protein, g | 48 | 53 | 12 |
|  |  |  |  | Carbohydrates, g | 379 | 416 | 10 |
|  |  |  |  | Total fat, g | 24 | 29 | 17 |
|  |  |  |  | Vitamin A RAE, μg | 210 | 307 | 46 |
|  |  |  |  | Vitamin D, μg | 1.1 | 1.2 | 18 |
|  |  |  |  | Vitamin E, mg | 4.3 | 5.1 | 19 |
|  |  |  |  | Thiamine, mg | 0.7 | 0.9 | 14 |
|  |  |  |  | Riboflavin, mg | 0.4 | 0.5 | 25 |
|  |  |  |  | Niacin, mg | 13.4 | 15 | 11 |
|  |  |  |  | Vitamin B6 | 1.1 | 1.3 | 18 |
|  |  |  |  | Folate, μg | 115 | 148 | 29 |
|  |  |  |  | Vitamin C, mg | 41 | 62 | 52 |
|  |  |  |  | Calcium, mg | 260 | 324 | 25 |
|  |  |  |  | Iron, mg | 9.4 | 11.3 | 20 |
|  |  |  |  | Sodium, mg | 4096 | 5538 | 35 |
|  |  |  |  | Potassium, mg | 1329 | 1646 | 24 |
|  |  |  |  | Magnesium, mg | 306 | 354 | 16 |
|  |  |  |  | Zinc, mg | 8.2 | 9.2 | 12 |
| Lividini *et al.* (2013) ^(40)^;  Rajshahi, Bangladesh;  medians | 2 non-consecutive days of 12-hour observed weighed food records & 12-hour dietary recall (n=477) | 14-day food diary of  households with children age 2-3 (n=513) | WRA  (n=240) | Energy, kcal | 1984 | 2281 | 13 |
| Lividini *et al.* (2013) ^(40)^;  Dhaka, Bangladesh;  medians | 2 non-consecutive days of 12-hour observed weighed food records & 12-hour dietary recall (n=464) | 14-day food diary of  households with children age 2-3 (n=678) | WRA  (n=238) | Energy, kcal | 1693 | 2329 | 27 |
| ***II. Study compared consumption of fortified food vehicles derived from individual-level versus HCES dietary data*** | | | | | | | |
| Engle-Stone & Brown (2015) ^(18)^;  Cameroon;  medians | 24H individual recall  (N=1794) | Combination of 3 to 7 day recalls and 15-day diary depending on sub-population, different survey sample  (n=4363) | WRA  (n=912) | Refined oil, g | 19.6 | 13.3 | -47 |
|  |  |  |  | Wheat flour, g | 76.6 | 51.2 | -50 |
|  |  |  |  | Sugar, g | 29.2 | 15.5 | -88 |
|  |  |  |  | Bouillon, g | 1.9 | 2.7 | 30 |
| Dary & Jariseta (2012) ^(11)^;  Kampala, Uganda;  medians | 24H individual recall | 7-day recall of household meals  (n=314) | WRA (15-49 years) | Vegetable oil, g | 10.4 | 12.8 | 19 |
|  |  |  |  | Wheat flour, g | 58.3 | 29.3 | -99 |
|  |  |  |  | Sugar, g | 55.5 | 45.6 | -22 |
|  |  |  |  | Maize flour, g | 115.9 | 44.4 | -161 |
|  |  |  |  | Rice, g | 63.0 | 37.5 | -68 |
| Dary & Jariseta (2012) ^(11)^;  Southwestern Region, Uganda;  medians | 24H individual recall | 7-day recall of household meals  (n=322) | WRA (15-49 years) | Vegetable oil, g | 10.9 | 9.1 | -20 |
|  |  |  |  | Wheat flour, g | 35.3 | 13.7 | -158 |
|  |  |  |  | Sugar, g | 40.3 | 21.2 | -90 |
|  |  |  |  | Maize flour, g | 78.6 | 49.4 | -59 |
|  |  |  |  | Rice, g | 81.3 | 33.1 | -146 |
| Dary & Jariseta (2012) ^(11)^;  Northern Region, Uganda;  medians | 24H individual recall | 7-day recall of household meals  (n=321) | WRA (15-49 years) | Vegetable oil, g | 18.8 | 7.0 | -169 |
|  |  |  |  | Wheat flour, g | 35.6 | 6.1 | -484 |
|  |  |  |  | Sugar, g | 39.4 | 12.3 | -220 |
|  |  |  |  | Maize flour, g | 130.3 | 19.6 | -565 |
|  |  |  |  | Rice, g | 84.4 | 17.7 | -377 |

# **Appendix 1.** Original protocol of systematic review developed according to PRISMA guidelines

[Original protocol attached as a separate document]

[PRISMA checklist attached as a separate document]

# **Appendix 2.** Search algorithms returning publications in database search

| Database | Search Algorithm |
| --- | --- |
| Web of Science | TOPIC: (hces) Timespan: All years. Indexes: SCI-EXPANDED, SSCI, A&HCI, CPCI-S, CPCI-SSH, ESCI.  TOPIC: (household consumption and expenditure survey) Timespan: All years. Indexes: SCI-EXPANDED, SSCI, A&HCI, CPCI-S, CPCI-SSH, ESCI.  TOPIC: (lsms) Timespan: All years. Indexes: SCI-EXPANDED, SSCI, A&HCI, CPCI-S, CPCI-SSH, ESCI.  TOPIC: ("living standards measurement study") Timespan: All years. Indexes: SCI-EXPANDED, SSCI, A&HCI, CPCI-S, CPCI-SSH, ESCI.  TOPIC: ("integrated household survey") Timespan: All years. Indexes: SCI-EXPANDED, SSCI, A&HCI, CPCI-S, CPCI-SSH, ESCI.  TOPIC: ("household budget survey") Timespan: All years. Indexes: SCI-EXPANDED, SSCI, A&HCI, CPCI-S, CPCI-SSH, ESCI.  TOPIC: ("living conditions monitoring survey") Timespan: All years. Indexes: SCI-EXPANDED, SSCI, A&HCI, CPCI-S, CPCI-SSH, ESCI.  TOPIC: ("socioeconomic survey") Timespan: All years. Indexes: SCI-EXPANDED, SSCI, A&HCI, CPCI-S, CPCI-SSH, ESCI.  TOPIC: ("national risk and vulnerability assessment") Timespan: All years. Indexes: SCI-EXPANDED, SSCI, A&HCI, CPCI-S, CPCI-SSH, ESCI.  TOPIC: ("core welfare indicators") Timespan: All years. Indexes: SCI-EXPANDED, SSCI, A&HCI, CPCI-S, CPCI-SSH, ESCI.  TOPIC: ("enquête" AND "auprès des ménages") Timespan: All years. Indexes: SCI-EXPANDED, SSCI, A&HCI, CPCI-S, CPCI-SSH, ESCI.  TOPIC: ("enquête" AND "les conditions de vie") Timespan: All years. Indexes: SCI-EXPANDED, SSCI, A&HCI, CPCI-S, CPCI-SSH, ESCI.  TOPIC: ("enquête" AND "le budget et la consommation des ménages") Timespan: All years. Indexes: SCI-EXPANDED, SSCI, A&HCI, CPCI-S, CPCI-SSH, ESCI. |
| PubMed | Search: “household consumption and expenditure survey” AND “food consumption”  Search: “household consumption and expenditure survey” AND nutr*  Search: "integrated household survey"  Search: "living standards measurement study"  Search: "living conditions monitoring survey" AND “food consumption”  Search: "living conditions monitoring survey" AND nutr*  Search: "national risk and vulnerability assessment"  Search: "core welfare indicators questionnaire" |
| AgEcon | Query : "household consumption and expenditure survey"  Query : "living standards measurement study"  Query : “household budget survey” AND nutr*  Query : “household budget survey” AND vitamin  Query : “household budget survey” AND mineral  Query : “household budget survey” AND micronutrient  Query : “household budget survey” AND energy intake  Query : “income and expenditure survey” AND nutr*  Query : “income and expenditure survey” AND vitamin  Query : “income and expenditure survey” AND mineral  Query : “income and expenditure survey” AND micronutrient  Query : “income and expenditure survey” AND energy intake  Query : "living conditions monitoring survey"  Query : "integrated household survey" |
| GARDIAN CGIAR | household consumption and expenditure survey  living standards measurement study  “integrated household survey” AND nutri*  living conditions monitoring survey  household budget survey |
| AGRIS | Query : "household consumption and expenditure survey"  Query : "living standards measurement study"  Query : “household budget survey” AND nutri*  Query : “household budget survey” AND energy  Query : “household budget survey” AND vitamin  Query : “household budget survey” AND mineral  Query : “household budget survey” AND micronutrient  Query : “income and expenditure survey” AND nutri*  Query : “income and expenditure survey” AND energy  Query : “income and expenditure survey” AND vitamin  Query : “income and expenditure survey” AND mineral  Query : “income and expenditure survey” AND micronutrient  Query : “integrated household survey” |
| JSTOR | ((“household consumption and expenditure survey”) AND (nutr*))  ((("living standards measurement study") AND (food)) AND (consumption))  (("living standards measurement study") AND (nutr*))  ((("integrated household survey") AND (food)) AND (consumption))  ((("integrated household survey") AND (nutr*)))  ((("integrated household survey") AND (micronutrient)))  ((“living conditions monitoring survey”) AND (nutr*)) |
| IFPRI Publications & Tools Database | household consumption and expenditure survey  living standards measurement study  integrated household survey  living conditions monitoring survey  household budget survey  socioeconomic survey  national risk and vulnerability assessment |
| Academic Search Complete | household consumption and expenditure survey  integrated household survey  living standards measurement study  household budget survey  living conditions monitoring survey  household income and expenditure survey |
| EconLit | (household consumption and expenditure survey).mp. [mp=heading words, abstract, title, country as subject]  (living standards measurement study).mp. [mp=heading words, abstract, title, country as subject]  (integrated household survey).mp. [mp=heading words, abstract, title, country as subject]  (living conditions monitoring survey).mp. [mp=heading words, abstract, title, country as subject]  (socioeconomic survey).mp. [mp=heading words, abstract, title, country as subject]  (national risk and vulnerability assessment).mp. [mp=heading words, abstract, title, country as subject]  (core welfare indicators questionnaire).mp. [mp=heading words, abstract, title, country as subject] |
| Global Health Database | (household consumption and expenditure survey).mp. [mp=abstract, title, original title, broad terms, heading words, identifiers, cabicodes]  living standards measurement study.mp. [mp=abstract, title, original title, broad terms, heading words, identifiers, cabicodes]  integrated household survey.mp. [mp=abstract, title, original title, broad terms, heading words, identifiers, cabicodes]  living conditions monitoring survey.mp. [mp=abstract, title, original title, broad terms, heading words, identifiers, cabicodes]  (household budget survey AND nutri*).mp. [mp=abstract, title, original title, broad terms, heading words, identifiers, cabicodes]  socioeconomic survey.mp. [mp=abstract, title, original title, broad terms, heading words, identifiers, cabicodes] |
| Global Index Medicus | search: household consumption AND expenditure survey  search: tw:("living standards measurement study")  search: integrated household survey  search: living conditions monitoring survey  search: tw:(socioeconomic survey AND (energy OR calor*)) AND (la:("en")) AND (year_cluster:[2000 TO 2020]) |
| Scopus | TITLE-ABS-KEY (household AND consumption AND expenditure AND survey AND nutri*)  TITLE-ABS-KEY ("living standards measurement study")  TITLE-ABS-KEY (household AND budget AND survey AND nutri*)  TITLE-ABS-KEY (living AND conditions AND monitoring AND survey)  TITLE-ABS-KEY (integrated AND household AND survey AND nutri*) |
| BASE | search: "household consumption and expenditure survey"  search: "living standards measurement study" AND *nutri*  search: "integrated household survey" AND *nutri*  search: "living conditions monitoring survey"  search: household budget survey AND *nutri*  search: "socioeconomic survey" AND *nutri* |
| Google Scholar | search: "Enquête" AND "nutrition" AND "auprès des ménages"  search: "Enquête" AND "nutrition" AND "les conditions de vie" AND "auprès des ménages"  search: "Enquête" AND "nutrition" AND "Suivi de la Pauvrete"  search: "Enquête" AND "nutrition" AND " la Consommation et le Secteur Informel"  search: "Enquête” AND "nutrition" AND "le Budget et la Consommation des Menages" |

# **Appendix 3.** Information extracted from the included literature

| **Categories** | **Extracted information** |
| --- | --- |
| General information | - Title - Year published - Authors - Journal/publication medium - Peer review (Y/N) - Objective - Reported principal findings - Reported limitations of analysis |
| Question 1: What is the scope of the existing literature? | Scope   - Country(s) of study - Name of survey - Data collection time period - Households, N - Dietary data collection method - Which nutrients were measured (or energy)?   Quality   - Recall period - Food item list, n - Survey question verb - Conversion to standard units? - Edible portions of foods? How? - Outliers identified? How? - Which food composition tables were used and in what priority? |
| Question 2: What metrics have been used to estimate nutrient and energy supply/apparent intake and nutrient adequacy from HCES data, and how those estimates compare across metrics? | - What nutrition metric was used? - Household or individual level? - Was energy requirement calculation necessary for metric? If yes:   - What was the physical activity level parameter?   - What was the body weight parameter?   - Account for pregnancy? How?   - Account for lactation? How? - Did analysis measure inadequacy?   - What method was used to determine inadequacy?   - Was dietary reference value necessary? Which was used? - For certain nutrients, account for bioavailability? |
| Question 3: How do HCES-derived estimates of apparent nutrient and energy intake compare to those estimated using individual-level dietary assessment methods? Do results differ by age group (i.e. adults, adolescents, children, infants)? | - Name of HCES - Data collection time period - Households, N - Individual dietary assessment methods   - Participants, n   - Members of households?   - Retrospective or prospective?   - Number of visits - Apparent nutrient/energy intake estimate from HCES - Nutrient/energy intake estimate from individual dietary assessment - Difference in nutrient/energy intake vs. apparent nutrient/energy intake - Measured inadequacy? - Difference in inadequacy prevalence between HCES and individual assessments - Disaggregated by age groups?   - Difference in nutrient/energy intake vs. apparent nutrient/energy intake by age group - Disaggregated by gender?   - Difference in nutrient/energy intake vs. apparent nutrient/energy intake by sex |
| Question 4: What types of sub-group comparative analyses have been conducted using nutrient and energy supply/apparent intake estimates from HCES data? (PROGRESS+ framework) | Disaggregation of results by:   - Place of residence - Race/ethnicity - Occupation - Gender - Religion - Education - Socioeconomic status - Social capital - + (personal characteristics) |

References

1. Ambagna JJ & Dury S (2016) De la disponibilité à la consommation alimentaire: analyse des évolutions de la consommation alimentaire à l’échelle nationale et des ménages au Cameroun. *10 èmes Journées Rech. en Sci. Soc.*

2. Beegle K, De Weerdt J, Friedman J, et al. (2012) Methods of household consumption measurement through surveys: Experimental results from Tanzania. *J. Dev. Econ.* **98**, 3–18.

3. Bell W, Coates JC, Rogers BL, et al. (2019) Getting the food list ‘right’: An approach for the development of nutrition-relevant food lists for household consumption and expenditure surveys. *Public Health Nutr.* **22**, 246–256.

4. Bermudez OI, Lividini K, Smitz MF, et al. (2012) Estimating micronutrient intakes from Household Consumption and Expenditures Surveys (HCES): an example from Bangladesh. *Food Nutr. Bull.* **33**, S208-13.

5. Bogard JR, Marks GC, Mamun A, et al. (2017) Non-farmed fish contribute to greater micronutrient intakes than farmed fish: Results from an intra-household survey in rural Bangladesh. *Public Health Nutr.* **20**, 702–711.

6. Bogard JR, Farook S, Marks GC, et al. (2017) Higher fish but lower micronutrient intakes: Temporal changes in fish consumption from capture fisheries and aquaculture in Bangladesh. *PLoS One* **12**, e0175098.

7. Bromage S, Rosner B, Rich-Edwards JW, et al. (2018) Comparison of methods for estimating dietary food and nutrient intakes and intake densities from household consumption and expenditure data in Mongolia. *Nutrients* **10**, 703.

8. Chakrabarti S, George N, Majumder M, et al. (2018) Identifying sociodemographic, programmatic and dietary drivers of anaemia reduction in pregnant Indian women over 10 years. *Public Health Nutr.* **21**, 2424–2433.

9. Chege CGK, Andersson CIM & Qaim M (2015) Impacts of Supermarkets on Farm Household Nutrition in Kenya. *World Dev.* **72**, 394–407.

10. D’Souza A & Tandon S (2015) *Using Household and Intrahousehold Data To Assess Food Insecurity: Evidence From Bangladesh*. *ERR-190*. Washington, D.C..

11. Dary O & Jariseta ZR (2012) Validation of dietary applications of Household Consumption and Expenditures Surveys (HCES) against a 24-hour recall method in Uganda. *Food Nutr. Bull.* **33**, S190-8.

12. De Weerdt J, Beegle K, Friedman J, et al. (2014) *The Challenge of Measuring Hunger*. *Policy Res. Work. Pap.* Washington, DC.

13. DeFries R, Chhatre A, Davis KF, et al. (2018) Impact of Historical Changes in Coarse Cereals Consumption in India on Micronutrient Intake and Anemia Prevalence. *Food Nutr. Bull.* **39**, 377–392.

14. Donovan C & Massingue J (2007) Illness, death, and macronutrients: Adequacy of rural Mozambican household production of macronutrients in the face of HIV/AIDS. *Food Nutr. Bull.* **28**, S331-8.

15. Dop MC, Pereira C, Mistura L, et al. (2012) Using Household Consumption and Expenditures Survey (HCES) data to assess dietary intake in relation to the nutrition transition: a case study from Cape Verde. *Food Nutr. Bull.* **33**, S221-7.

16. Ecker O & Qaim M (2011) Analyzing Nutritional Impacts of Policies: An Empirical Study for Malawi. *World Dev.* **39**, 412–428.

17. Ecker O & Qaim M (2008) Income and Price Elasticities of Food Demand and Nutrient Consumption in Malawi. In *Am. Agric. Econ. Assoc. Annu. Meet.*, p. 6349. Orlando, Florida: American Agricultural Economics Association.

18. Engle-Stone R & Brown KH (2015) Comparison of a household consumption and expenditures survey with nationally representative food frequency questionnaire and 24-hour dietary recall data for assessing consumption of fortifiable foods by women and young children in Cameroon. *Food Nutr. Bull.* **36**, 211–30.

19. Engle-Stone R, Sununtnasuk C & Fiedler JL (2017) Investigating the significance of the data collection period of household consumption and expenditures surveys for food and nutrition policymaking: Analysis of the 2010 Bangladesh household income and expenditure survey. *Food Policy* **72**, 72–80.

20. Euler M, Krishna V, Schwarze S, et al. (2017) Oil Palm Adoption, Household Welfare, and Nutrition Among Smallholder Farmers in Indonesia. *World Dev.* **93**, 219–235.

21. Fiedler JL (2014) Food crop production, nutrient availability, and nutrient intakes in Bangladesh: Exploring the agriculture-nutrition nexus with the 2010 Household Income and Expenditure Survey. *Food Nutr. Bull.* **35**, 487–508.

22. Fiedler JL, Babu S, Smitz MF, et al. (2012) Indian social safety net programs as platforms for introducing wheat flour fortification: A case study of Gujarat, India. *Food Nutr. Bull.* **33**, 11–30.

23. Fiedler JL & Lividini K (2014) Managing the vitamin A program portfolio: A case study of Zambia, 2013-2042. *Food Nutr. Bull.* **35**, 105–25.

24. Fiedler JL, Lividini K & Bermudez OI (2015) Estimating the impact of vitamin A-fortified vegetable oil in Bangladesh in the absence of dietary assessment data. *Public Health Nutr.* **18**, 414–20.

25. Fiedler JL, Lividini K, Drummond E, et al. (2016) Strengthening the contribution of aquaculture to food and nutrition security: The potential of a vitamin A-rich, small fish in Bangladesh. *Aquaculture* **452**, 291–303.

26. Fiedler JL, Lividini K, Kabaghe G, et al. (2013) Assessing Zambia’s industrial fortification options: Getting beyond changes in prevalence and cost-effectiveness. *Food Nutr. Bull.* **34**, 501–19.

27. Fiedler JL, Lividini K, Zulu R, et al. (2013) Identifying Zambia’s industrial fortification options: Toward overcoming the food and nutrition information gap-induced impasse. *Food Nutr. Bull.* **34**, 480–500.

28. Gilbert R, Benson T & Ecker O (2019) *Are Malawian diets changing? An assessment of nutrient consumption and dietary patterns using household-level evidence from 2010/11 and 2016/17*. *MaSSP Work. Pap. 30*. Washington, DC.

29. Hall C, Macdiarmid JI, Matthews RB, et al. (2019) The relationship between forest cover and diet quality: a case study of rural southern Malawi. *Food Secur.* **11**, 635–650.

30. Hirvonen K, Taffesse AS & Worku Hassen I (2016) Seasonality and household diets in Ethiopia. *Public Health Nutr.* **19**, 1723–1730.

31. Hjelm L, Mathiassen A & Wadhwa A (2016) Measuring Poverty for Food Security Analysis: Consumption-Versus Asset-Based Approaches. *Food Nutr. Bull.* **37**, 275–289.

32. Imhoff-Kunsch B, Flores R, Dary O, et al. (2012) Methods of using household consumption and expenditures survey (HCES) data to estimate the potential nutritional impact of fortified staple foods. *Food Nutr. Bull.* **33**, S185-9.

33. Jariseta ZR, Dary O, Fiedler JL, et al. (2012) Comparison of estimates of the nutrient density of the diet of women and children in Uganda by Household Consumption and Expenditures Surveys (HCES) and 24-hour recall. *Food Nutr. Bull.* **33**, S199-207.

34. Jati IRAP, Vadivel V, Nöhr D, et al. (2012) Nutrient density score of typical Indonesian foods and dietary formulation using linear programming. *Public Health Nutr.* **15**, 2185–92.

35. Johnecheck WA & Holland DE (2007) Nutritional status in postconflict Afghanistan: Evidence from the National Surveillance System Pilot and National Risk and Vulnerability Assessment. *Food Nutr. Bull.* **28**, 3–17.

36. Joy EJM, Kumssa DB, Broadley MR, et al. (2015) Dietary mineral supplies in Malawi: spatial and socioeconomic assessment. *BMC Nutr.* **1**, 42.

37. Kankwamba H & Kornher L (2019) Household shocks, infrastructural investments, food and nutrition security linkages in Malawi. In *93rd Annu. Conf. Agric. Econ. Soc.* Coventry, UK: Agricultural Economics Society.

38. Karageorgou D, Imamura F, Zhang J, et al. (2018) Assessing dietary intakes from household budget surveys: A national analysis in Bangladesh. *PLoS One* **13**, e0202831.

39. Lividini K & Fiedler JL (2015) Assessing the promise of biofortification: A case study of high provitamin A maize in Zambia. *Food Policy* **54**, 65–77.

40. Lividini K, Fiedler JL & Bermudez OI (2013) Policy implications of using a Household Consumption and Expenditures Survey versus an Observed-Weighed Food Record Survey to design a food fortification program. *Food Nutr. Bull.* **34**, 520–32.

41. Marivoet W, De Herdt T & Ulimwengu J (2018) *Eviter les écueils statistiques de la RDC – nouvelles estimations sur les tendances du bien-être et de la pauvreté (2005-2012) selon une approche de désagrégation spatiale*. *Univ. Antwerp- Inst. Dev. Policy Work. Pap.* Antwerp.

42. Mathiassen A & Hollema S (2014) What is the effect of physical activity level on food consumption, energy deficiency, and dietary diversity? *Food Nutr. Bull.* **35**, 351–360.

43. Mishra V & Ray R (2009) Dietary diversity, food security and undernourishment: The vietnamese evidence. *Asian Econ. J.* **23**, 225–247.

44. Molini V (2006) *Food Security in Vietnam During the 1990s: The Empirical Evidence*. *Hunger Food Secur. New Challenges New Oppor.* Amsterdam.

45. Mwangi DM, Fiedler JL & Sununtnasuk C (2016) *Imputing nutrient intake from foods prepared and consumed away from home and other composite foods: Exploring extensions of the Subramanian–Deaton cost per calorie approach*. *IFPRI Discuss. Pap.* Washington, D.C..

46. Nguyen MC & Winters P (2011) The impact of migration on food consumption patterns: The case of Vietnam. *Food Policy* **36**, 71–87.

47. Rahman M (2012) Estimating the Effects of Social Safety Net Programmes in Bangladesh on Calorie Consumption of Poor Households. *Bangladesh Dev. Stud.* **35**, 67–85.

48. Rose D & Charlton KE (2002) Quantitative Indicators from a Food Expenditure Survey Can Be Used to Target the Food Insecure in South Africa. *J. Nutr.* **132**, 3235–3242.

49. Tiwari S, Skoufias E & Sherpa M (2013) *Shorter, Cheaper, Quicker, Better: Linking Measures of Household Food Security to Nutritional Outcomes in Bangladesh, Nepal, Pakistan, Uganda, and Tanzania*. *Policy Res. Work. Pap. Ser.* Washington, D.C..

50. Smith LC, Alderman H & Aduayom D (2006) *Food insecurity in Sub-Saharan Africa: New estimates from household expenditure surveys*. *Res. Rep.* Washington, D.C..

51. Smith MR, DeFries R, Chhatre A, et al. (2019) Inadequate Zinc Intake in India: Past, Present, and Future. *Food Nutr. Bull.* **40**, 26–40.

52. Tandon S & Landes R (2010) The Sensitivity of Food Security in India. *Econ. Polit. Wkly.* **46**, 92–99.

53. Trinh Thi H, Simioni M & Thomas-Agnan C (2018) Decomposition of changes in the consumption of macronutrients in Vietnam between 2004 and 2014. *Econ. Hum. Biol.* **31**, 259–275.

54. Troubat N & Grünberger K (2017) Impact of survey design in the estimation of habitual food consumption: A study based on urban households of Mongolia. *Food Policy* **72**, 132–145.

55. Ulimwengu J, Liverpool-Tasie S, Randriamamonjy J, et al. (2011) *Understanding the linkage between agricultural productivity and nutrient consumption: evidence from Uganda.* *IFPRI - Discuss. Pap.* Washington, D.C..

56. Verduzco-Gallo I, Ecker O & Pauw K (2014) *Changes in Food and Nutrition Security in Malawi Analysis of Recent Survey Evidence*. *MaSSP Work. Pap. 6*. Washington, D.C..

57. Weinberger K (2003) The impact of micronutrients on labor productivity: Evidence from rural India. In *25th Int. Conf. Agric. Econ.* Durban, South Africa: International Association of Agricultural Economists.

58. Worku IH, Dereje M, Minten B, et al. (2017) Diet transformation in Africa: the case of Ethiopia. *Agric. Econ.* **48**, 73–86.
